# Integrating bulk and single-cell sequencing reveals cellular heterogeneity between lung adenocarcinoma in smokers and never-smokers

**DOI:** 10.7555/JBR.39.20250160

**Published:** 2026-05-21

**Authors:** Zihuan Zhao, Pan Yang, Yuhan Liu, Kai Wang, Xianfeng Xu, Yuzhuo Wang, Meng Zhu, Na Qin, Cheng Wang, Weimin Li, Hongxia Ma, Zhoufeng Wang, Hongbing Shen

**Affiliations:** 1 Department of Epidemiology, Center for Global Health, School of Public Health, Nanjing Medical University, Nanjing, Jiangsu 211166, China; 2 Institute of Respiratory Health, Frontiers Science Center for Disease-related Molecular Network, West China Hospital, Sichuan University, Chengdu, Sichuan 610041, China; 3 Department of Epidemiology, School of Public Health, Southeast University, Nanjing, Jiangsu 210096, China; 4 Research Units of Cohort Study on Cardiovascular Diseases and Cancers, Chinese Academy of Medical Sciences, Beijing 100037, China

**Keywords:** lung cancer in smokers, lung cancer in never-smokers, single-cell RNA sequencing, cellular heterogeneity

## Abstract

Lung cancer in smokers (LCIS) and lung cancer in never-smokers (LCINS) are different entities with distinct molecular features. However, their cellular heterogeneity still requires further investigation. Through an integrated analysis of single-cell RNA sequencing and bulk sequencing data, we identified cell subpopulations associated with smoking and non-smoking patients. Subsequent transcriptomic analyses were performed to elucidate differences in cellular functions and the tumor microenvironment. We observed that smoking-associated cancer cells exhibited a higher degree of aggressiveness, which may correlate with an adverse prognosis in smoking patients. Additionally, immunosuppressive *CXCL10*^+^ macrophages may contribute to tumorigenesis in smokers, and the immunoregulatory LGALS9-HAVCR2 axis could be a potential immunotherapeutic target. In non-smokers, the inflammatory microenvironment may be involved in tumor development. Moreover, the reduced anti-tumor cytotoxicity may be associated with their suboptimal immunotherapeutic response. Our study uncovered differences in oncogenic and immune escape mechanisms between LCIS and LCINS patients and suggests potential immunotherapeutic strategies.

## Introduction

Lung cancer is the leading cause of cancer-related mortality worldwide^[1]^. Despite the strong association between lung cancer development and cigarette smoke, nearly 25% of lung cancer cases, predominantly lung adenocarcinoma (LUAD), occur in individuals who have never smoked^[2]^. With the decline in smoking rates, the proportion of lung cancer in never-smokers (LCINS) has increased and was estimated to be the fifth leading cause of cancer-related deaths in 2023^[3–4]^. LCINS preferentially affects females and individuals of East Asian descent^[3]^, and it exhibits a comparatively better prognosis than lung cancer in smokers (LCIS)^[5]^. Although several risk factors have been found to be associated with lung cancer in lifelong non-smokers, including radon exposure, hormonal influences, and genetic factors^[2]^, the exact pathogenesis of LCINS remains unclear.

Accumulating evidence has shown that LCIS and LCINS exhibit distinct genomic and transcriptomic features^[3]^. Previous studies have shown that non-smoking patients have a lower tumor mutational burden than smoking patients^[6]^. Moreover, 78%–92% of non-smoking patients carry driver gene alterations, while only 49.5% of smoking-related LUAD patients harbor driver gene mutations^[6]^. Specifically, *EGFR* is the most common driver gene mutation in LCINS, with a prevalence ranging from 60% to 74% among non-smoking female lung cancer patients of East Asian ethnicity^[7]^. *TP53* and *KRAS* driver gene mutations are more prevalent in smoking patients^[8]^. Transcriptomic studies have demonstrated distinct gene expression profiles and functional pathway activities between smokers and non-smokers. Smoking patients display increased expression of cell cycle-related genes and enrichment of the *p53* pathway, whereas non-smokers and long-term former smokers show lower stemness scores^[9]^. However, these findings are primarily derived from bulk sequencing, which only reflects averaged gene expression, thereby making it challenging to understand intratumoral heterogeneity^[10]^.

The advent of single-cell RNA sequencing (scRNA-seq) technology has enabled the comparison of cellular composition across different phenotypes, the identification of cell subpopulations associated with specific phenotypes, and the exploration of cellular interaction differences^[11–12]^. For example, Martos *et al*^[13]^ discovered a dysfunctional natural killer (NK)-like CD8^+^ T cell subtype enriched in smokers, while Duclos *et al*^[14]^ demonstrated that smoking might be linked to the loss of club cells and an increased number of goblet cells. A recent study comparing scRNA-seq data from smoking and non-smoking LUAD patients revealed that cancer cells in smoking patients exhibited greater aggressiveness, while the immunosuppressive tumor microenvironment might promote tumor growth in non-smoking patients^[5]^.

However, these scRNA-seq studies typically involve small sample sizes, which limit the statistical power to correlate phenotypes with specific cell subtypes reliably. Recently, researchers have developed SCISSOR^[11]^, a bioinformatics algorithm that leverages phenotype information from large bulk cohorts to identify cell subpopulations most relevant to disease phenotypes. By utilizing this algorithm, a previous study discovered that LUAD patients with the *EGFR* mutation were associated with dendritic cell (DC) infiltration, while those with *TP53* and *STK11* mutations exhibited higher CD8^+^ T cell infiltration^[15]^.

In the current study, to elucidate the cellular heterogeneity between smoking and non-smoking LUAD patients, we incorporated data from 140 LUAD patients who underwent bulk RNA sequencing from the Nanjing-Huaxi Lung Cancer Consortium (NHLCC) and 92553 cells from 20 East Asian LUAD patients who underwent scRNA-seq. By integrating scRNA-seq and bulk sequencing data using the SCISSOR algorithm, we explored the cellular-level differences between smoking and non-smoking patients. Our results offer new insights into the distinct tumor microenvironment of smoking and non-smoking patients and provide potential treatment strategies.

## Materials and methods

### Study population

In our study, we used bulk RNA-sequencing data from 140 LUAD patients enrolled in the NHLCC cohort. Specifically, 78 patients were from the Nanjing Lung Cancer Cohort (NJLCC), and 62 were from West China Hospital. Each sample was assessed and examined by at least one pathologist to validate the histopathological attributes and tumor cell content, with only tumor samples containing a minimum of 70% tumor cells included. The study was approved by the Institutional Review Board of Nanjing Medical University (Nanjing, Jiangsu, China; project identification code: 2018.848) and West China Hospital (Chengdu, Sichuan, China; project identification code: 2017.114). The study was performed in accordance with the Declaration of Helsinki, and all participants provided written informed consent. The scRNA-seq data were downloaded from the Single-cell Lung Cancer Atlas (https://luca.icbi.at)^[15]^. A total of 92553 primary LUAD tumor cells from 20 East Asian LUAD patients were extracted using the 10X Genomics platform. Baseline patient characteristics of the bulk sequencing and scRNA-seq datasets are shown in ***Supplementary Tables 1*** and ***2***, respectively.

### Bulk RNA sequencing data preprocessing

Total RNA was extracted using the Qiagen RNeasy kit, following the manufacturer's instructions. The standard Illumina RNA-seq protocol (http://support.illumina.com/training/online-courses/sequencing.html) was employed to produce transcription profiles of these samples, using the Illumina HiSeq 1500 platform. Subsequently, the raw RNA-seq reads were mapped to the GENCODE v19 genome assembly using STAR v2.4.1 with default parameters^[16]^. To mitigate technical variability, raw counts underwent ComBat-seq batch correction using the sva package (v3.38.0) with default parameters (group = NULL, covar_mod = NULL) to retain biological variation while adjusting for batch effects^[17]^. Before correction, counts were normalized to counts per million to account for sequencing depth differences. Bulk RNA-seq gene expression data and clinical annotations of The Cancer Genome Atlas Lung Adenocarcinoma (TCGA-LUAD) cohort were retrieved from UCSC Xena (https://xena.ucsc.edu/), and the survival information was obtained from previous research^[18]^. A total of 498 patients with complete tumor staging and survival data were retained for downstream analysis.

### Processing and annotation of scRNA data

The Seurat package (v4.0.1) was used for the scRNA-seq data processing^[19]^. All ribosomal genes were excluded from subsequent analyses. Raw counts were normalized using the 'NormalizeData' function. Because the scRNA-seq data came from two studies, the highly variable genes in each dataset were determined using the 'FindVariableFeatures' function, and the common highly variable genes were identified using the 'SelectIntegrationFeatures' function. Batch effects were addressed through Seurat's reciprocal principal component analysis-based integration workflow, which generated batch-corrected expression matrices by identifying integration anchors across batches using the 'FindIntegrationAnchors' function and then merging them using the 'IntegrateData' function. Batch correction was not applied to cancer cells to avoid masking biological variations. Principal component analysis, uniform manifold approximation and projection (UMAP) visualization, neighborhood analysis, and clustering analysis were performed using the 'RunPCA', 'RunUMAP', 'FindNeighbors', and 'FindClusters' functions from the Seurat package, respectively^[19]^. The identities of each cluster of major immune cell types were further annotated using marker genes identified by the 'FindAllMarkers' function.

### Identification of smoking-associated and non-smoking-associated cell subgroups

The SCISSOR algorithm was applied to integrate bulk RNA-seq and scRNA-seq data to identify smoking-associated (Scissor_S) and non-smoking-associated (Scissor_NS) cell subpopulations in LUAD^[11]^. Briefly, a correlation matrix was first computed between each bulk sample and single-cell sample, based on common gene expression using Pearson correlation. A hybrid approach, combining L1-regularization (Lasso) and network-based penalization, was implemented on the correlation matrix, with the balance between sparsity and network coherence controlled by the parameter alpha. The parameter alpha was tuned within a default search range (0.05–0.90), terminating when the selected cells were less than 20% of the total. For each fixed alpha, the regularization strength λ of the L1 model was chosen using cross-validation with the minimum averaged error. The reliability of the Scissor_S/NS classification was validated by comparing the proportions of Scissor_S/NS cells in Scissor-selected cells, and we found that cells from smoking and non-smoking patients had higher proportions of Scissor_S cells and Scissor_NS cells, respectively (***Supplementary Fig. 1***).

### Differential gene expression and functional enrichment analysis

Differential gene expression analysis of scRNA-seq data was performed using Seurat's 'FindMarkers' function (v4.0.1) with default parameters, including the Wilcoxon rank sum test, to compare batch-corrected normalized counts across groups, except for cancer cells, which retained uncorrected normalized counts as previously detailed. Genes with an absolute fold change (FC) greater than 1.25 and a false discovery rate (FDR) less than 0.05 were considered significantly differentially expressed genes. Gene set enrichment analysis (GSEA) was performed to assess pathway activity using the fgsea (v1.16.0) package^[20]^. Genes were ranked based on fold change, and gene sets were obtained from the Molecular Signatures Database (MsigDB)^[21–22]^. Gene sets with *P* < 0.05 and FDR < 0.25 were considered significantly enriched.

### Single sample gene set enrichment analysis (ssGSEA)

To assess functional differences among different cell subgroups, the ssGSEA method was employed to calculate the enrichment scores of known gene sets with specific biological significance^[23]^. The alveolar epithelial type Ⅱ cell (AT2) gene set was composed of known lung epithelial AT2 cell marker genes. The effector cell gene set consisted of cytotoxic T and NK cell marker genes, representing the level of T/NK cell infiltration^[24]^. The gene sets "immune suppression by myeloid cells", "checkpoint molecules", and "MHC-Ⅱ" comprised genes related to myeloid cell-mediated immune suppression, immune checkpoint molecules, and major histocompatibility complex (MHC)-Ⅱantigen presentation, respectively, as described in the previous study^[24]^.

### Signature construction and survival analysis

The differentially expressed genes of the Scissor-selected cells were ranked based on FC values, and a gene signature was constructed using the top 50 genes with the highest FC values. Bulk sample signature scores were calculated using the ssGSEA algorithm^[23]^. Patients were separated into high-score and low-score groups based on the median value of the signature score. Kaplan-Meier survival analysis and the log-rank test were performed to assess the survival differences. Additionally, a multivariate Cox regression analysis, adjusting for tumor stage, was conducted to investigate whether the gene signature represented an independent risk factor.

### Trajectory and cell-cell communication analysis

We used Monocle 3 for single-cell trajectory analysis to examine the differentiation differences between Scissor_S and Scissor_NS cancer cells^[25]^. To establish the differentiation trajectory, we included all cancer cells, along with normal AT2 and basal epithelial cells within the tumor samples, as these two cell types were recognized as the origins of lung cancer cells^[26–28]^, and were designated as root cells. The normalization and clustering of all included cells were conducted as previously described, and normal epithelial cells were designated as root cells. The pseudotime values of Scissor_S and Scissor_NS cancer cells were compared using the two-sided Wilcoxon rank sum test.

Intercellular communication in the scRNA-seq data was computed and inferred using CellChat (v2.1.2)^[12]^. The manually curated CellChatDB database was used as the reference. Normalized counts were used for CellChat analysis, and the core functions 'computeCommunProb' and 'computeCommunProbPathway' were employed to calculate the communication probabilities and identify significant signaling pathways. Additionally, the 'aggregateNet' function was used to analyze the communication network by tallying and summarizing the interaction counts and communication probabilities.

### Statistical analysis

For group comparisons, the Wilcoxon rank sum test was conducted for continuous variables, while the chi-square test and Fisher's exact test were employed for categorical variables. All reported *P* values were two-sided, with statistical significance defined as *P* < 0.05. *P* values were adjusted by FDR in multiple comparisons. Statistical analyses were performed using R software (v4.0.3), and all figures were generated using Seurat (v4.0.1), EnhancedVolcano (v1.12.0), ggplot2 (v3.3.5), and ggpubr (v0.4.0).

## Results

### Clinical characteristics and the study design

Among the 140 patients who underwent bulk sequencing, 47 were smokers (34%), and 93 were never-smokers (66%). The detailed clinical information is presented in ***Supplementary Table 1***. No significant differences were observed in clinical stage or age distribution between smoking and non-smoking patients. All smoking patients were male, while most non-smoking patients were female (70/93, 75%). Cox regression analysis revealed significantly poorer survival among smoking patients after adjusting for tumor stage (hazard ratio [HR] = 5.25, 95% confidence interval [CI]: 1.66–16.63, *P* = 0.005) (***Supplementary Fig. 2***). The clinical baseline information of the 20 LUAD patients undergoing scRNA-seq is detailed in ***Supplementary Table 2***. Fisher's exact test also revealed differences in sex distribution between smoking and non-smoking patients, with no significant differences observed in tumor stage or study origin.

**Figure 1:**
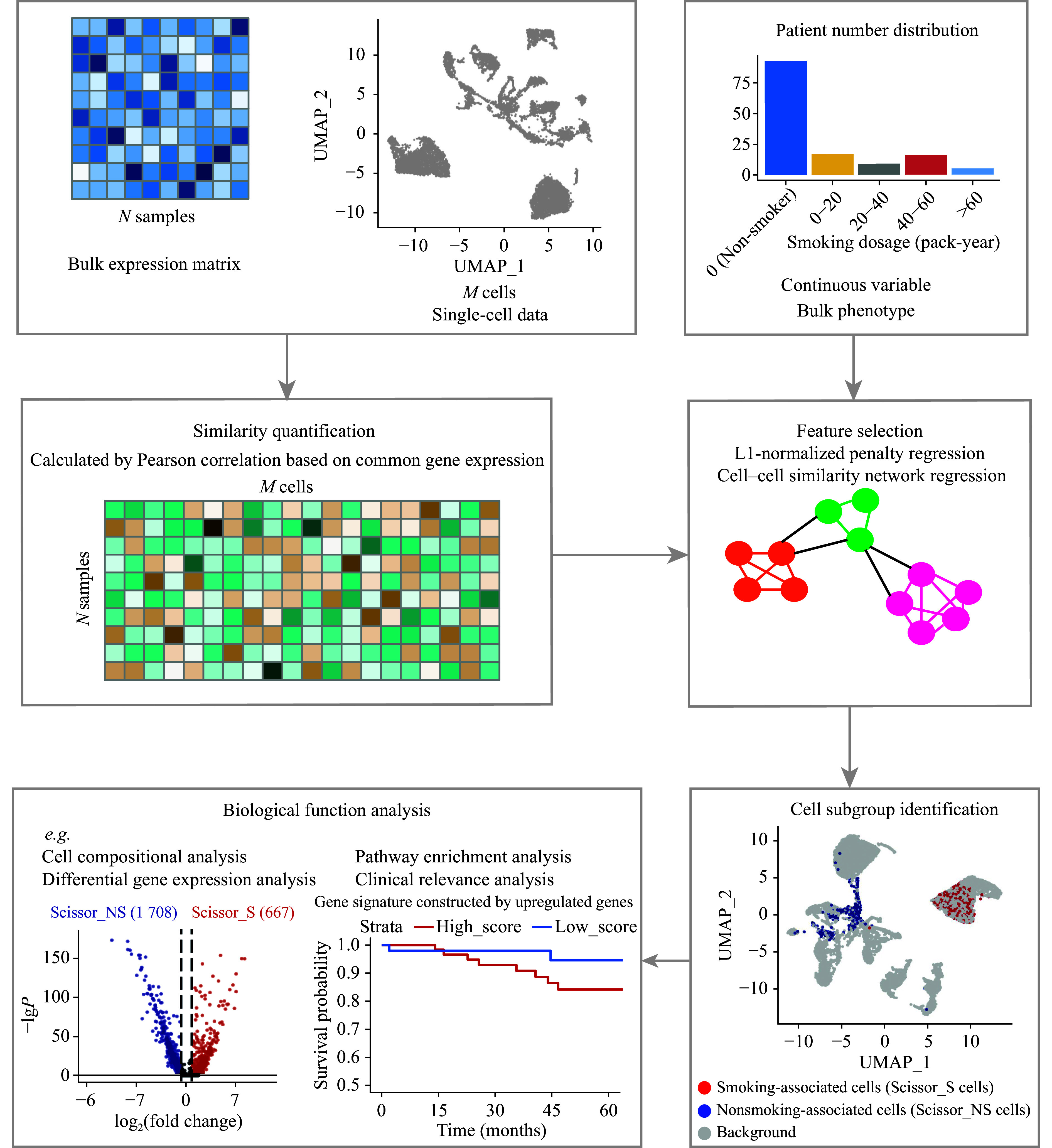
Overview of study workflow. Step 1: Similarity quantification. A correlation matrix was generated using Pearson correlation based on shared gene expression between bulk and single-cell datasets. Step 2: Identification of smoking-associated (Scissor_S) and non-smoking-associated (Scissor_NS) cell subpopulations. The Scissor algorithm was employed to integrate bulk and single-cell datasets. Using 140 bulk RNA-seq samples with smoking dosage information, LASSO regression and network-constrained models were applied to identify Scissor_S and Scissor_NS cell subpopulations within the scRNA-seq dataset. Step 3: Biological function analysis. Compositional and transcriptomic analyses were conducted on Scissor_S and Scissor_NS cell subpopulations. In addition, we explored the clinical relevance of these Scissor-selected cell subgroups. Abbreviations: Scissor_NS, non-smoking-associated subpopulations (by the Scissor algorithm); Scissor_S, smoking-associated subpopulations (by the Scissor algorithm); UMAP, uniform manifold approximation and projection.

The study workflow is shown in ***Fig. 1***. Initially, we defined smoking-associated (Scissor_S) cells and non-smoking-associated (Scissor_NS) cells using the Scissor algorithm. Next, we compared compositional and transcriptomic differences to uncover the underlying biological distinctions. Subsequently, we investigated the clinical relevance of Scissor-selected cells to identify potential prognostic markers and therapeutic strategies.

### Smoking-associated and non-smoking-associated cell distribution

A total of 92553 cells from 20 LUAD patients were included in the current study, with 51797 cells derived from smoking patients (56%) and 40756 from non-smoking patients (44%). Epithelial cells, CD4^+^ T cells, CD8^+^ T/NK cells, macrophages, and DCs were the predominant cell populations, together accounting for over 80% of cells in all LUAD patients, as well as in both LCIS and LCINS subgroups (***Supplementary Fig. 3A*** and ***3B***). A direct comparison of cell proportions between 13 smoking and 7 non-smoking patients was performed, using linear regression with the dataset as the covariate. No significant difference was observed (***Supplementary Fig. 3C***), possibly due to the limited sample size. Therefore, we used the Scissor algorithm, guided by 140 bulk RNA-sequencing samples, to identify smoking-associated and non-smoking-associated cell subpopulations.

**Figure 3:**
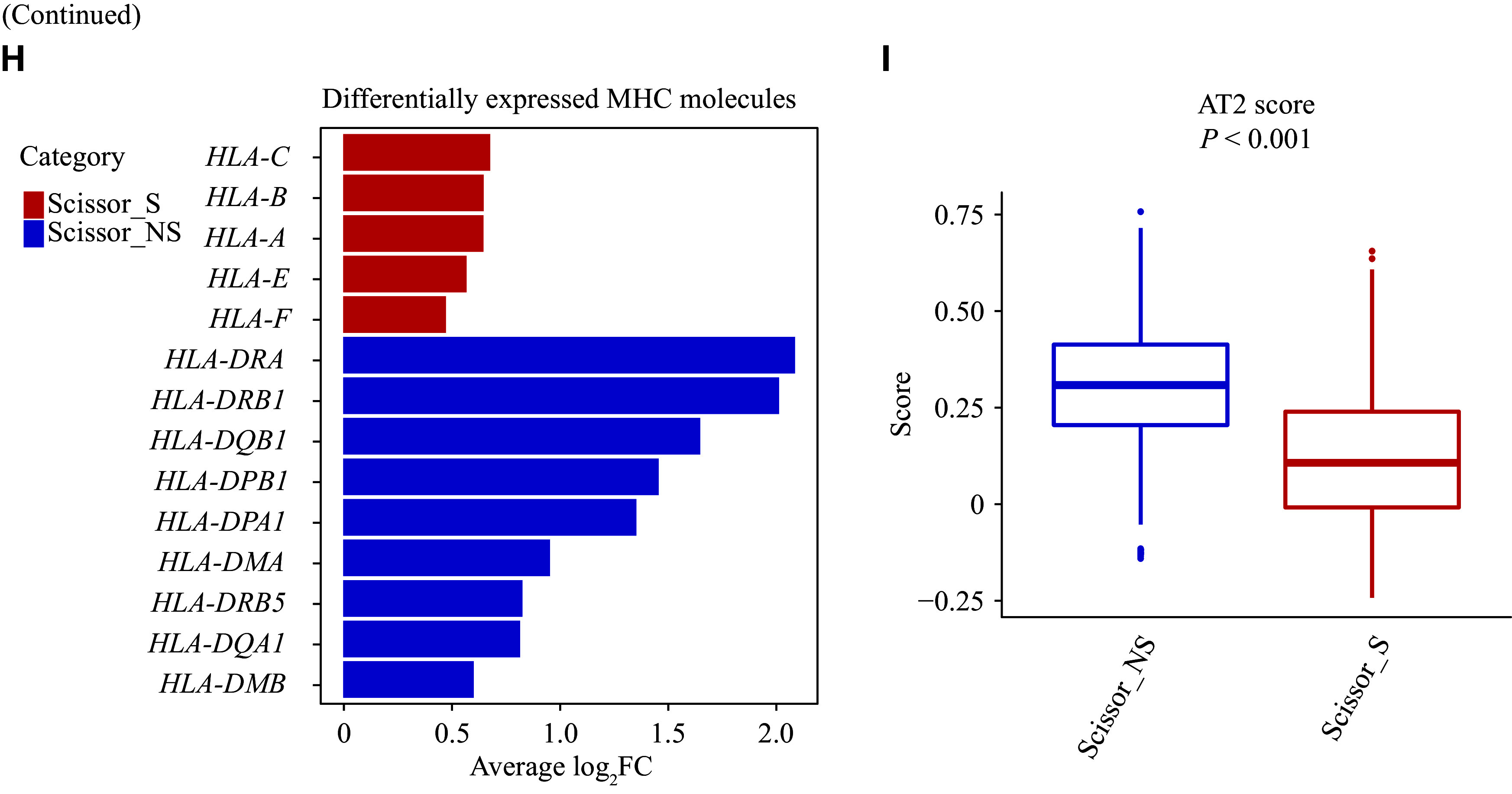
Characterization of smoking-associated and non-smoking-associated cancer cell subpopulations. A: UMAP plot of Scissor-selected smoking-associated and non-smoking-associated cancer cells. B: Volcano plot of differentially expressed genes between Scissor_S and Scissor_NS cancer cells (|FC| > 1.25 and FDR < 0.05). C: Bar plot of gene set enrichment analysis showing the top 10 pathways with the lowest FDR values in Scissor_S and Scissor_NS cancer cells, respectively. D: Comparison of pseudotime values between Scissor_S and Scissor_NS cancer cells. The *P* value was derived from the two-sided Wilcoxon test. E: Proportions of Scissor_S and Scissor_NS cancer cells from early or late clinical stages. The *P* value was derived from the two-tailed chi-squared test. F: Kaplan-Meier survival curves comparing high and low Scissor_S gene signature subgroups in the NHLCC cohort. The *P* value was derived from the two-tailed log-rank sum test and multivariate Cox regression adjusting for tumor stage. G: Kaplan-Meier survival curves comparing high and low Scissor_S gene signature subgroups in the TCGA-LUAD cohort. The *P* value was derived from the two-tailed log-rank sum test and multivariate Cox regression adjusting for tumor stage. H: Differentially expressed MHC molecules in Scissor_S and Scissor_NS cancer cells. I: Comparison of AT2 scores between Scissor_S and Scissor_NS cancer cells. The *P* value was derived from a two-sided Wilcoxon test. Abbreviations: AT2, alveolar epithelial type Ⅱ cell; CI: confidence interval; FC, fold change; FDR, false discovery rate; HR, hazard ratio; MHC, major histocompatibility complex; NHLCC, Nanjing-Huaxi Lung Cancer Consortium; TCGA-LUAD, The Cancer Genome Atlas Lung Adenocarcinoma; Scissor_S, smoking-associated subpopulations (by the Scissor algorithm); Scissor_NS, non-smoking-associated subpopulations (by the Scissor algorithm); UMAP, uniform manifold approximation and projection.

**Figure 2:**
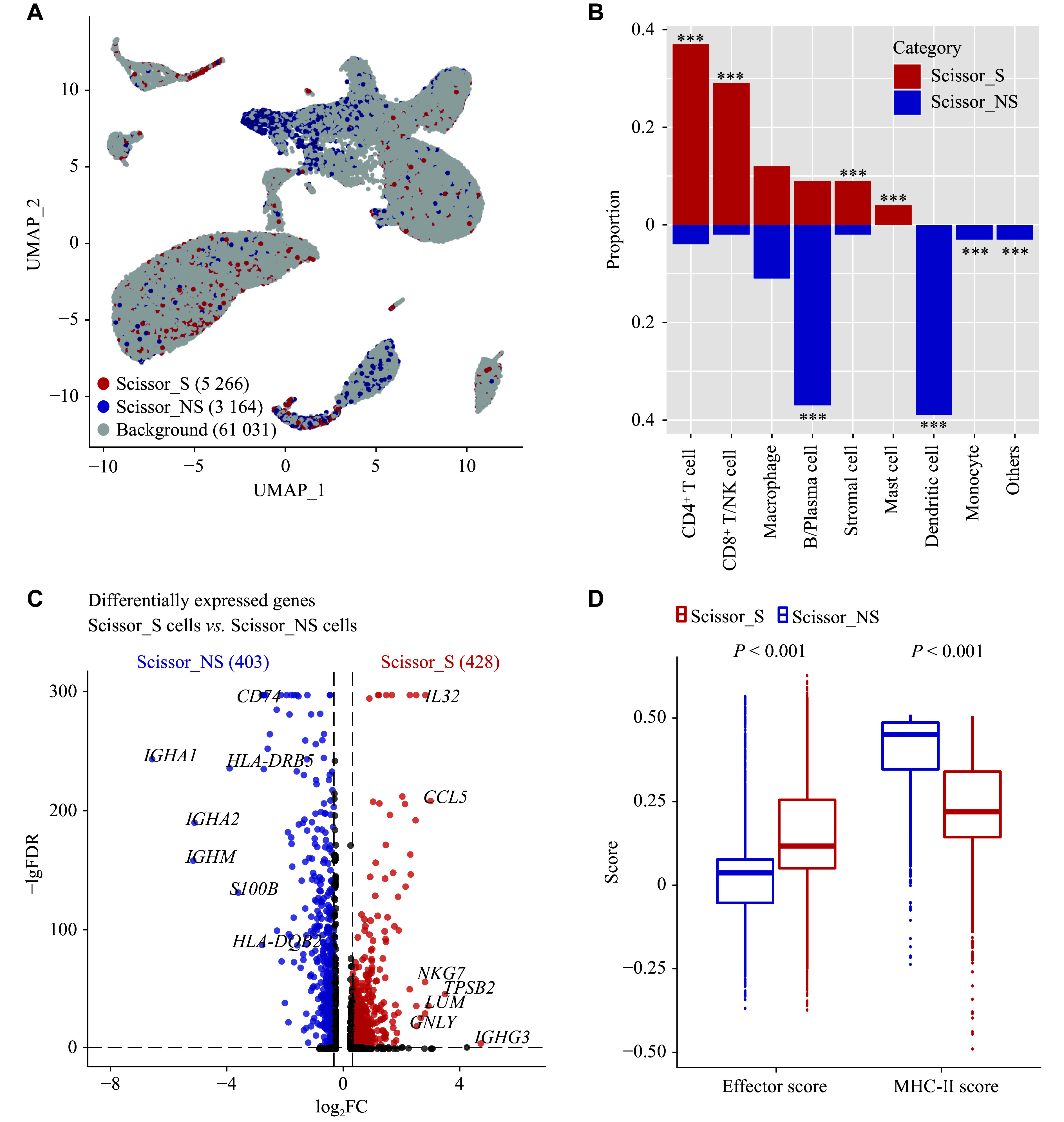
Smoking-associated and non-smoking-associated cell landscape. A: UMAP plot of Scissor-selected smoking-associated and non-smoking-associated cells. B: Comparison of cell subtype proportions in Scissor_S and Scissor_NS cells. ^***^FDR < 0.001. C: Volcano plot of differentially expressed genes between Scissor_S and Scissor_NS cells (|FC| > 1.25 and FDR < 0.05). D: Boxplot showing the MHC-Ⅱ score and effector cell score in Scissor_S and Scissor_NS cells. *P* values were calculated by the two-tailed Wilcoxon rank sum test. Abbreviations: FC, fold change; FDR, false discovery rate; MHC, major histocompatibility complex; Scissor_NS, non-smoking-associated subpopulations (by the Scissor algorithm); Scissor_S, smoking-associated subpopulations (by the Scissor algorithm); UMAP, uniform manifold approximation and projection.

A total of 5266 Scissor_S cells and 3164 Scissor_NS cells were identified (***Fig. 2A***). CD4^+^ T cells (37% *vs.* 4%, FDR < 0.001) and CD8^+^ T/NK cells (29% *vs.* 2%, FDR < 0.001) were predominantly present in Scissor_S cells, while DCs constituted the primary cell type in Scissor_NS cells (0% *vs.* 39%, FDR < 0.001) (***Fig. 2B***). Differential gene expression analysis (FC > 1.25, FDR < 0.05) revealed that 428 and 403 genes were upregulated in Scissor_S and Scissor_NS cells, respectively (***Fig. 2C***). IgG-related genes (*e.g.*, *IGHG3*) were highly expressed in Scissor_S cells, whereas the expression of IgA-related genes (*e.g.*, *IGHA1* and *IGHA2*) was elevated in Scissor_NS cells, suggesting potential differences in the infiltration of antibody-producing plasma cells between smoking and non-smoking patients (***Fig. 2C*** and ***Supplementary Table 3***). Additionally, the expression of several cell cytotoxicity-related genes (*e.g.*, *NKG7* and *GNLY*) was elevated in Scissor_S cells. Scissor_NS cells, on the other hand, expressed several HLA class Ⅱ molecules (*e.g.*, *HLA-DQB2* and *HLA-DRB5*) at high levels (***Fig. 2C*** and ***Supplementary Table 3***).

To further evaluate potential differences in the expression of cytotoxicity-related molecules and MHC class Ⅱ genes between Scissor_S and Scissor_NS cells, we computed the enrichment scores for an effector gene set, including CD8^+^ T cell/NK cell marker genes, and an MHC class Ⅱ gene set in these two cell populations. The results revealed that the effector gene set score was elevated in Scissor_S cells (median, 0.12 *vs*. 0.04, *P* < 0.001), whereas the MHC-Ⅱ gene set score was increased in Scissor_NS cells (median, 0.22 *vs.* 0.45, *P* < 0.001) (***Fig. 2D***). These findings suggest that the tumor immune microenvironment of smokers may have a greater infiltration of cytotoxic cells, while non-smokers exhibit a more abundant presence of MHC class Ⅱ^+^ antigen-presenting cells.

### Higher aggressiveness of cancer cells in LCIS patients

Cancer cells exhibit significant heterogeneity within each individual. Using the Scissor algorithm, we selected 1796 cancer cells, including 979 Scissor_S cancer cells and 817 Scissor_NS cancer cells (***Fig. 3A***). Differential gene expression analysis revealed that 862 genes were upregulated in Scissor_S cells and 610 genes were upregulated in Scissor_NS cells (***Fig. 3B***). Several oncogenes, including *ZFAS1* and *ALDOA*, were found to be overexpressed in Scissor_S cancer cells^[29–30]^. GSEA revealed the activation of pathways related to endocytosis, axon guidance, and various cancer-related pathways, such as "Thyroid_cancer" and "Small_cell_lung_cancer", as well as oncogenic signaling pathways like WNT, MAPK, and p53, in Scissor_S cancer cells (***Fig. 3C*** and ***Supplementary Table 4***). Trajectory analysis was performed with AT2/basal cells designated as the root population to infer the pseudotime values of cancer cells (***Supplementary Fig. 4A***). Genes associated with lung epithelial cell differentiation, such as *SFTPC* and *SCGB3A1*^[31]^, were inversely correlated with pseudotime values (***Supplementary Fig. 4B***), indicating that higher pseudotime corresponds to the loss of cellular differentiation and increased aggressiveness. We found that most Scissor_S cancer cells were positioned at the terminal end of the differentiation trajectory (***Fig. 3A*** and ***Supplementary Fig. 4A***), exhibiting significantly higher pseudotime values (median, 38.52 *vs.* 20.47, *P* < 0.001) than Scissor_NS cancer cells (***Fig. 3D***), indicating a more advanced stage of malignant progression. Correspondingly, a large proportion of Scissor_S cancer cells were derived from late-stage patients (84%), while most Scissor_NS cancer cells were sourced from early-stage tumor patients (85%) (***Fig. 3E***).

**Figure 3:**
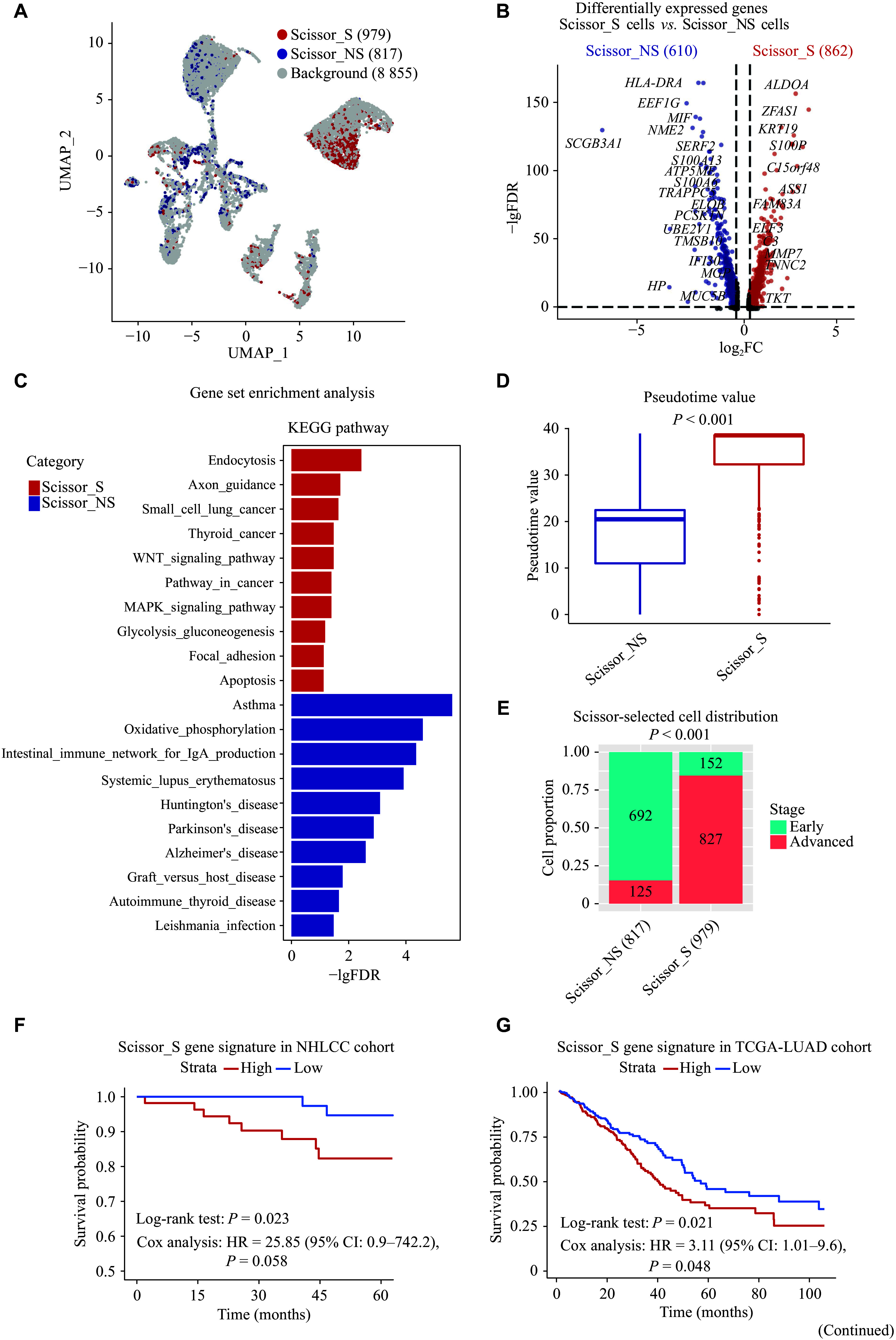


**Figure 4:**
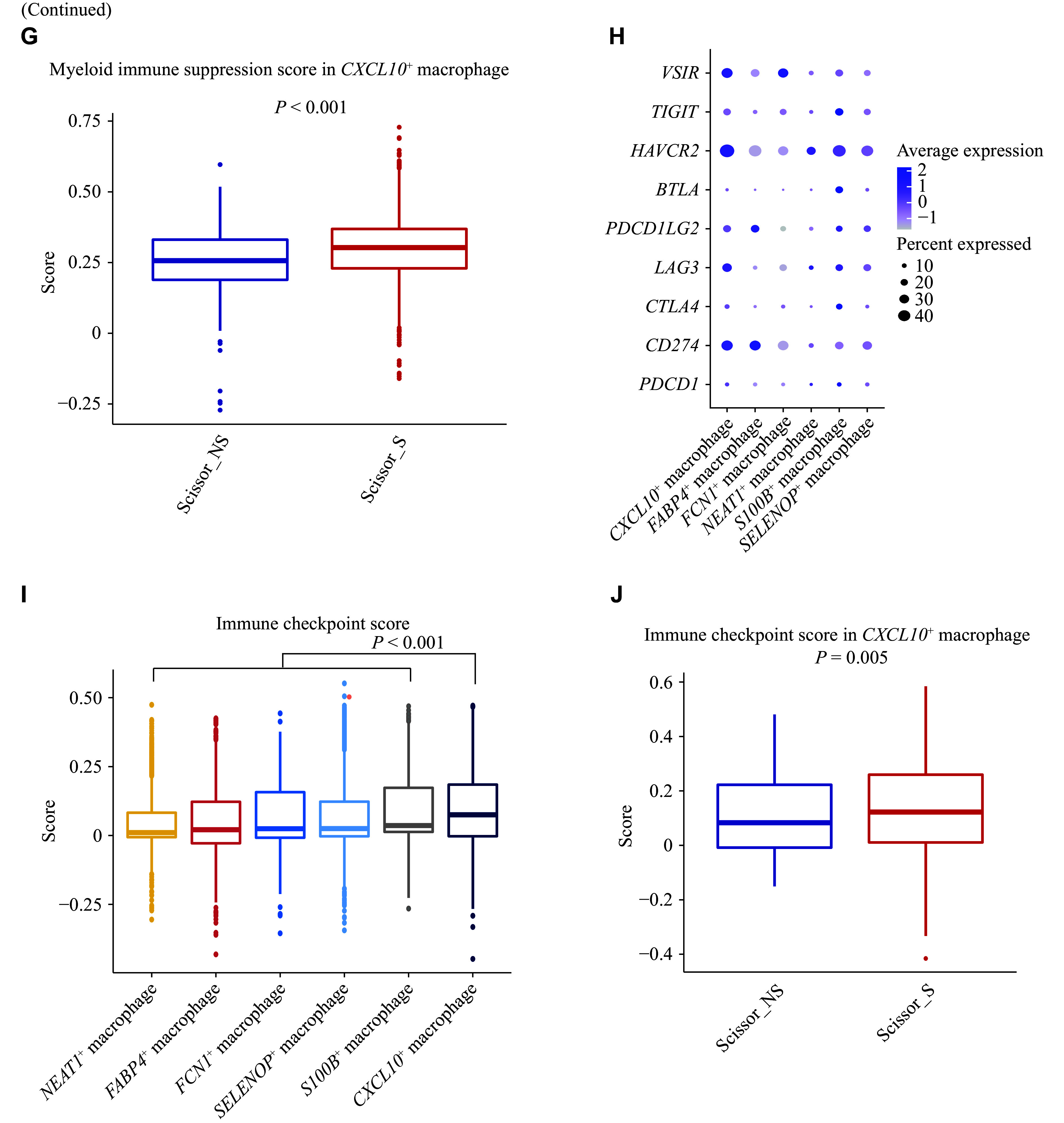
Characterization of smoking-associated and non-smoking-associated macrophage subpopulations. A: UMAP plot of macrophages, colored by cell subtypes. B: UMAP plot of Scissor-selected smoking-associated and non-smoking-associated macrophages. C: Comparison of cell subtype proportions between Scissor_S and Scissor_NS macrophages. ^***^FDR < 0.001. D: Volcano plot of differentially expressed genes between *CXCL10*^+^ macrophages and other macrophages (|FC| > 1.25 and FDR < 0.05). E: Bar plot of gene set enrichment analysis showing the top 10 pathways with the lowest FDR values in *CXCL10*^+^ macrophages and other macrophages, respectively. F: Myeloid immune suppression scores among different macrophage subtypes. The *P* value was derived from the two-sided Wilcoxon test comparison between *CXCL10*^+^ macrophages and other macrophages. G: Comparison of myeloid immune suppression scores between Scissor_S and Scissor_NS *CXCL10*^+^ macrophages. The *P* value was derived from the two-sided Wilcoxon test. H: Dot plot showing the expression of checkpoint genes in each macrophage subtype. I: Immune checkpoint score among different macrophage subtypes. The *P* value was derived from the two-sided Wilcoxon test comparison between *CXCL10*^+^ macrophages and other macrophages. J: Comparison of immune checkpoint scores between Scissor_S and Scissor_NS *CXCL10*^+^ macrophages. The *P* value was derived from the two-sided Wilcoxon test. Abbreviations: FC, fold change; FDR, false discovery rate; Scissor_S, smoking-associated subpopulations (by the Scissor algorithm); Scissor_NS, non-smoking-associated subpopulations (by the Scissor algorithm); UMAP, uniform manifold approximation and projection.

Subsequently, we selected the top 50 genes with the highest fold changes in Scissor_S cancer cells to construct the Scissor_S cancer cell signature and computed the signature score. Patients with higher expression levels of the Scissor_S gene signature had worse prognosis after adjusting for tumor stage in the NHLCC cohort (HR = 25.85, 95% CI: 0.9–742.2, *P* = 0.058) (***Fig. 3F***), and the association reached statistical significance in the TCGA-LUAD cohort (HR = 3.11, 95% CI: 1.01–9.60, *P* = 0.048) (***Fig. 3G***). Additionally, the normalized expression counts of MHC class Ⅱ molecules were elevated in Scissor_NS cancer cells, whereas MHC class Ⅰ molecules were found to be highly expressed in Scissor_S cancer cells (***Fig. 3H***). Furthermore, we observed a significantly higher expression of multiple AT2 cell markers (*e.g.*, *SFTPB* and *SFTPC*) in Scissor_NS cancer cells (***Supplementary Table 4***), along with a higher AT2 score (median, 0.11 *vs.* 0.31, *P* < 0.001) (***Fig. 3I***). AT2 cells play a key role in injury repair, and studies have shown that LUAD patients with higher AT2 scores have better prognoses^[5]^. Considering the established association between smoking and damage to the alveolar epithelium^[14]^, smoking-induced injury to AT2 cells may be linked to adverse outcomes in smokers.

Non-smoking-associated cancer cells showed elevated levels of inflammation-related molecules, such as *HP* and *MIF* (***Fig. 3B*** and ***Supplementary Table 4***). GSEA indicated that Scissor_NS cancer cells were correlated with inflammation-related diseases (*e.g.*, asthma) (***Fig. 3C***), suggesting a potential role of chronic inflammation in lung cancer development in never-smokers. Additionally, the oxidative phosphorylation pathway was elevated in Scissor_NS cancer cells, which may also be linked to the inflammatory tumor microenvironment^[32]^.

### Immunosuppressive property of smoking-associated macrophages

Macrophages play an intrinsic role in the tumor immune microenvironment and were classified into six subgroups (***Fig. 4A*** and ***Supplementary Fig. 5***). A total of 1437 Scissor_S macrophages and 1275 Scissor_NS macrophages were identified (***Fig. 4B***). The *CXCL10*^+^ macrophages were significantly overrepresented in Scissor_S cells (54% *vs.* 11%, FDR < 0.001), while the *NEAT1*^+^ macrophages were more prevalent among Scissor_NS macrophages (6% *vs.* 36%, FDR < 0.001) (***Fig. 4C***). A total of 158 genes were found to be overexpressed in the *CXCL10*^+^ macrophages (***Fig. 4D***). Among them, multiple types of cytokines were elevated in the *CXCL10*^+^ macrophages, including interleukins (*e.g.*, *IL4I1* and *IL1B*), chemokines (*e.g.*, *CXCL9* and *CXCL10*), and interferons (*e.g.*, *IFITM3* and *IFITM1*) (***Fig. 4D*** and ***Supplementary Table 5***). Pathway analysis also revealed that *CXCL10*^+^ macrophages were enriched in interleukin, cytokine, and interferon signaling pathways (***Fig. 4E***), indicating that they were involved in immune regulation in the tumor microenvironment. Within cytokine-related pathways, the *IL-10* signaling pathway was highly active in *CXCL10*^+^ macrophages. IL-10 is an anti-inflammatory cytokine that inhibits the activities of multiple immune cells^[33]^. Moreover, compared with other macrophages, *CXCL10*^+^ macrophages had a significantly higher "myeloid cell immune suppression" score (0.24 *vs.* 0.19, *P* < 0.001) (***Fig. 4F***). Additionally, the immune suppression score of Scissor_S *CXCL10*^+^ macrophages was significantly higher than that of Scissor_NS *CXCL10*^+^ macrophages (median, 0.30 *vs.* 0.26, *P* < 0.001) (***Fig. 4G***). Taken together, immunosuppressive, smoking-associated *CXCL10*^+^ macrophages may be involved in the carcinogenesis of smokers.

**Figure 4:**
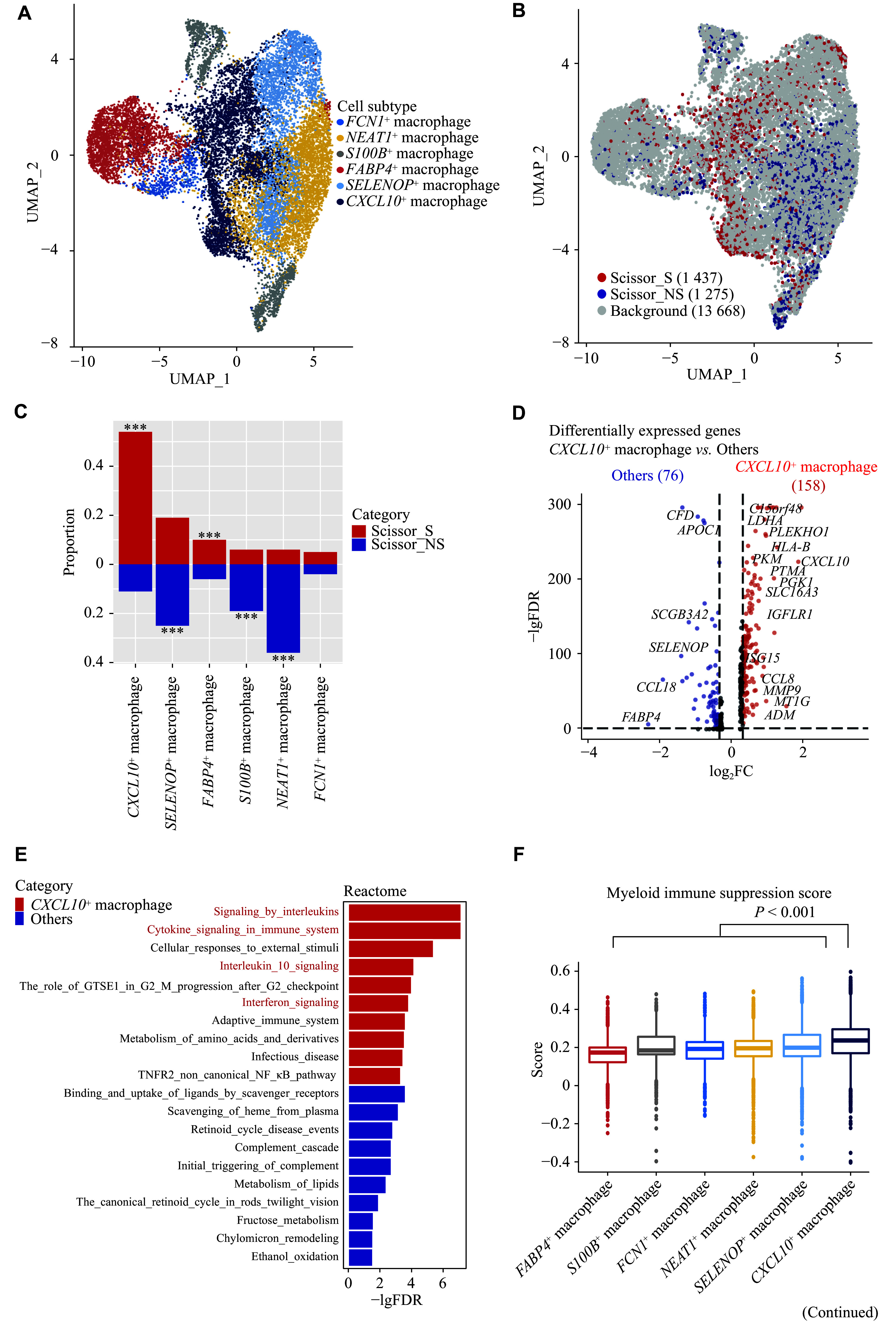


**Figure 5:**
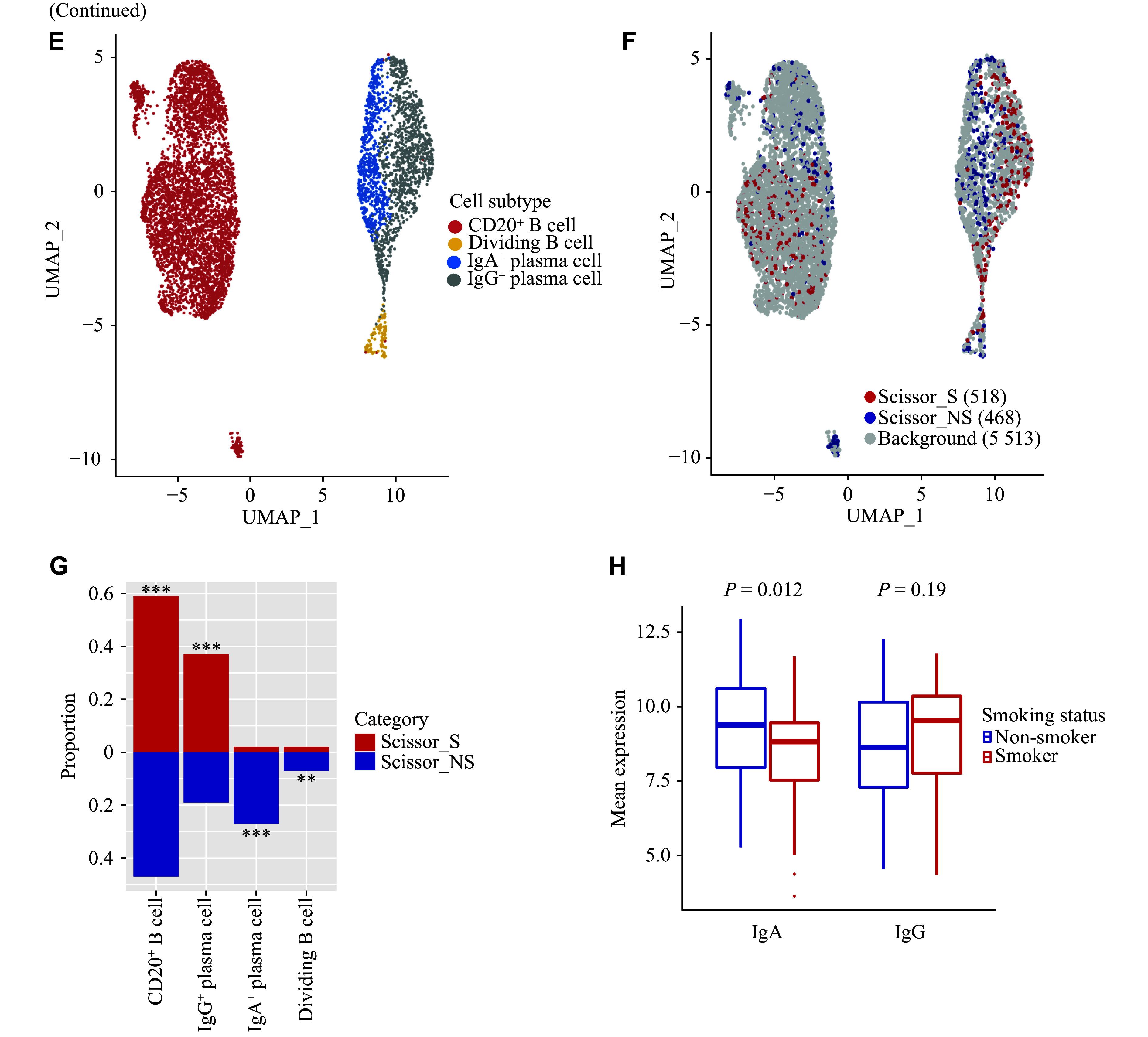
Characterization of smoking-associated and non-smoking-associated lymphocyte cell subpopulations. A: UMAP plot of CD8^+^ T/NK cells, colored by cell subtypes. B: UMAP plot of Scissor-selected smoking-associated and non-smoking-associated CD8^+^ T/NK cell subpopulations. C: Comparison of cell subtype proportions between Scissor_S and Scissor_NS CD8^+^ T/NK cells. ^**^FDR < 0.01 and ^***^FDR < 0.001. D: Volcano plot of differentially expressed genes between Scissor_S and Scissor_NS NK/CTL cells (|FC| > 1.25 and FDR < 0.05). E: UMAP plot of B/plasma cells, colored by cell subtypes. F: UMAP plot of Scissor-selected smoking-related and non-smoking-related B/plasma cell subpopulations. G: Comparison of cell subtype proportions between Scissor_S and Scissor_NS B/plasma cells. ^**^FDR < 0.01 and ^***^FDR < 0.001. H: Comparison of IgA- and IgG-related gene expression between smoking and non-smoking bulk RNA-seq patients. Abbreviations: CTL, cytotoxic T lymphocyte; FC, fold change; FDR, false discovery rate; NK, natural killer; Scissor_NS, non-smoking-associated subpopulations (by the Scissor algorithm); Scissor_S, smoking-associated subpopulations (by the Scissor algorithm); UMAP, uniform manifold approximation and projection.

*CXCL10* plays a critical role in recruiting cytotoxic CD8^+^ T cells to the tumor site^[34]^. Correlation analysis in bulk tissues also revealed a significant association between *CXCL10*^+^ macrophage infiltration and the expression of cytotoxic and exhausted T cell marker genes (*CD8A*, *GZMB*, *PRF1*, and *PDCD1*) (***Supplementary Fig. 6***). Additionally, although not reaching a significant threshold (FC > 1.25), several immune checkpoint molecules, including *PD-L1* (*CD274*), were overexpressed in *CXCL10*^+^ macrophages (***Fig. 4H***). They also exhibited a significantly higher immune checkpoint score (0.08 *vs.* 0.02, *P* < 0.001) (***Fig. 4I***). Furthermore, the checkpoint score of Scissor_S *CXCL10*^+^ macrophages was significantly higher than that of Scissor_NS macrophages (median, 0.12 *vs.* 0.08, *P* = 0.005) (***Fig. 4J***). Given that both cytotoxic cell infiltration and immune checkpoint molecule expression are predictive of immunotherapeutic efficacy, the enrichment of *CXCL10*^+^ macrophages may be associated with a favorable immunotherapy response in smoking patients.

*NEAT1* has been reported to be involved in the activation of the inflammasome in macrophages^[35]^. Additionally, multiple inflammation-related genes, such as *APOC1* and *IFI27*, were highly expressed in *NEAT1*^+^ macrophages. Pathway analysis revealed that *NEAT1*^+^ macrophages were enriched in the lysosome pathway (***Supplementary Table 5***). Therefore, the aggregation of *NEAT1*^+^ macrophages in the Scissor_NS macrophage population may contribute to the inflammatory microenvironment observed in non-smoking patients.

### Enrichment of cytotoxic plasmacytoid DCs in smoking-related DCs

DCs were classified into eight subgroups according to gene expression profiles (***Supplementary Figs. 7*** and ***8A***). We identified 358 Scissor_S DCs and 415 Scissor_NS DCs by the Scissor algorithm (***Supplementary Fig. 8B***). *CXCL10*^+^ DCs (38% *vs.* 7%, FDR < 0.001) and plasmacytoid DCs (23% *vs.* 0%, FDR < 0.001) were found to be enriched in Scissor_S DCs (***Supplementary Fig. 8C***). A total of 300 genes were found to be highly expressed in *CXCL10*^+^ DCs. Apart from *CXCL10*, multiple marker genes of monocytes, including *S100A9*, *S100A8*, and *CD14*, displayed the highest fold change values (***Supplementary Table 6***), suggesting that *CXCL10*^+^ DCs may originate from circulating monocytes. Compared with other types of DCs, plasmacytoid DCs expressed cytotoxic molecules such as *GZMB*, indicating their tumor elimination capability (***Supplementary Fig. 8D*** and ***Supplementary Table 6***). The most prevalent cell subtype of Scissor_NS DCs was the *CD1A*^+^ DC (4% *vs.* 32%, FDR < 0.001) (***Supplementary Fig. 8C***). *CD1A* plays a role in mediating antigen presentation to T cells^[36]^, and our results suggest that DCs in non-smoking patients exhibit a higher antigen presentation capability than those in smoking patients. Pathway enrichment analysis revealed distinct pathway activities for *CXCL10*^+^ DCs, plasmacytoid DCs, and *CD1A*^+^ DCs. For example, the extracellular matrix-related pathways were highly active in *CXCL10*^+^ DCs, while plasmacytoid DCs exhibited lower activity of the innate immune system and cytokine signaling pathways (***Supplementary Table 6***).

### Distinct lymphocyte anti-tumor activities in smoking and non-smoking patients

CD8^+^ T cells and NK cells are major cytotoxic effector cells, which were categorized into eight subgroups (***Fig. 5A*** and ***Supplementary Fig. 9***). Among them, NK cells and cytotoxic T lymphocytes (CTLs), collectively known as NK/CTL cells, expressed the highest levels of cytotoxic molecules (***Supplementary Fig. 9***). Using Scissor, we extracted 1763 CD8^+^ T/NK Scissor_S cells and 1162 CD8^+^ T/NK Scissor_NS cells (***Fig. 5B***). We observed that Scissor_S CD8^+^ T/NK cells had a higher fraction of cytotoxic T/NK cells (30% *vs.* 12%, FDR < 0.001), whereas the proportion of *GZMK*^+^CD8^+^ T cells was elevated in Scissor_NS cells (14% *vs.* 37%, FDR < 0.001) (***Fig. 5C***). *GZMK*^+^CD8^+^ T cells were reported to be less cytotoxic^[5]^, and compared with NK/CTL cells, *GZMK*^+^CD8^+^ T cells possessed lower levels of cytotoxic molecules (*e.g.*, *GNLY* and *PRF1*) (***Supplementary Fig. 9***). Pathway enrichment analysis revealed that multiple cytotoxicity-related pathways (*e.g.*, natural killer cell-mediated cytotoxicity) were activated in NK/CTL cells, but their activities were significantly decreased in *GZMK*^+^CD8^+^ T cells (***Supplementary Table 7***). Additionally, when comparing the transcriptomic differences between Scissor_S and Scissor_NS NK/CTL cells, we observed that the expression of cytotoxic molecules (*e.g.*, *FGFBP2*, *GZMB*, and *NKG7*) was downregulated in Scissor_NS NK/CTL cells (***Fig. 5D***). Our results indicated that non-smoking patients exhibited decreased cytotoxic T/NK cell infiltration, along with reduced activity.

**Figure 5:**
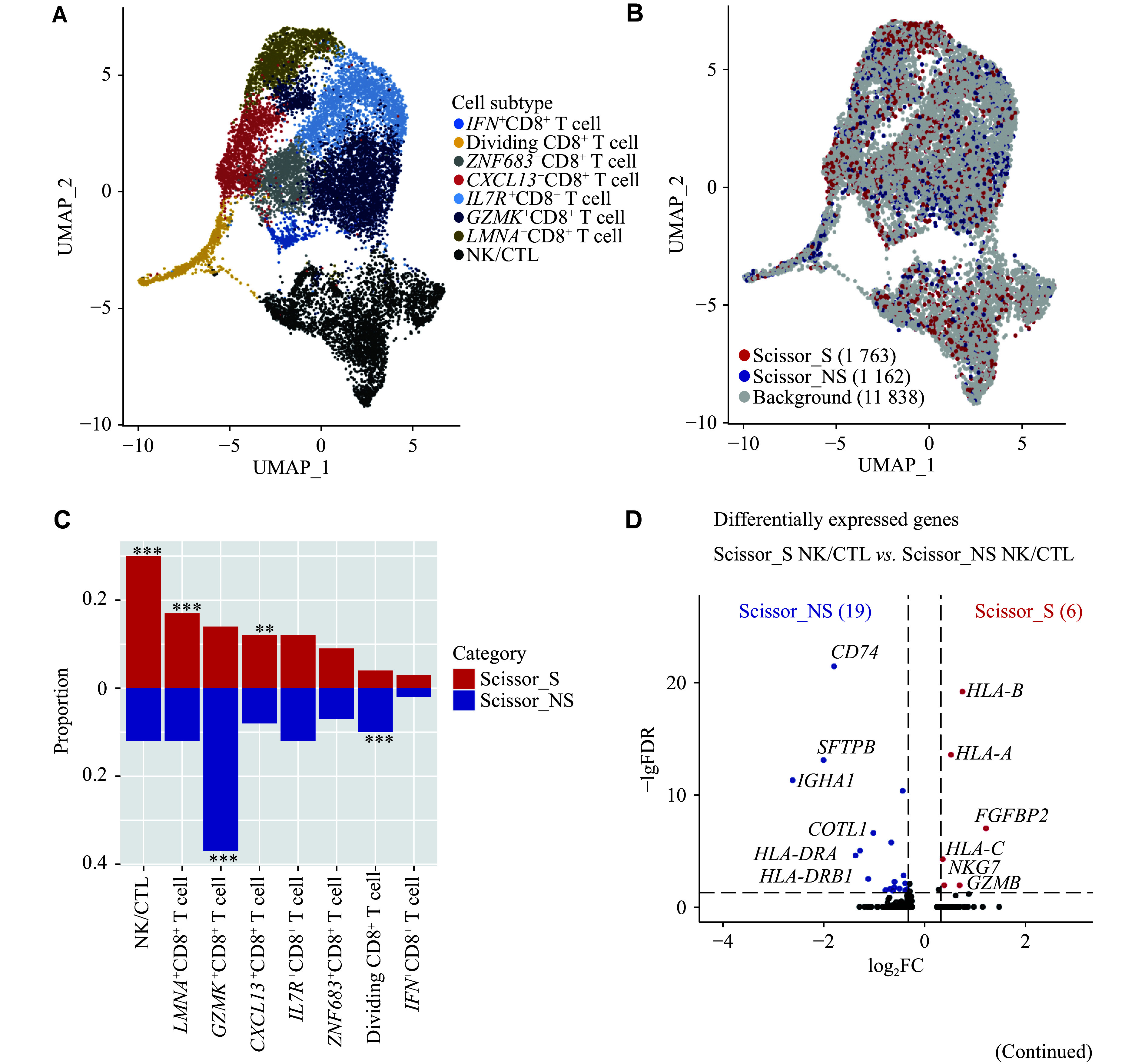


B/plasma cells represent a distinct type of lymphocyte that produces a significant amount of antibodies and were classified into CD20^+^ B cells, dividing B cells, IgA^+^ plasma cells, and IgG^+^ plasma cells (***Fig. 5E*** and ***Supplementary Fig. 10***). After applying Scissor, 518 Scissor_S cells and 468 Scissor_NS cells were identified (***Fig. 5F***). Scissor_S cells had a higher proportion of CD20^+^ B cells (59% *vs.* 47%, FDR < 0.001) and IgG^+^ plasma cells (37% *vs.* 19%, FDR < 0.001), whereas IgA^+^ plasma cells were predominantly found in Scissor_NS cells (2% *vs.* 27%, FDR < 0.001) (***Fig. 5G***). Concordantly, bulk tissue analysis revealed a significantly elevated expression of IgA in non-smokers (median value of mean IgA-related gene expression, 8.83 *vs.* 9.38, *P* = 0.012) (***Fig. 5H***). Our findings demonstrated variations in the infiltration of antibody-producing plasma cells between smoking and non-smoking patients.

### Cell-cell communication analysis

**Figure 6:**
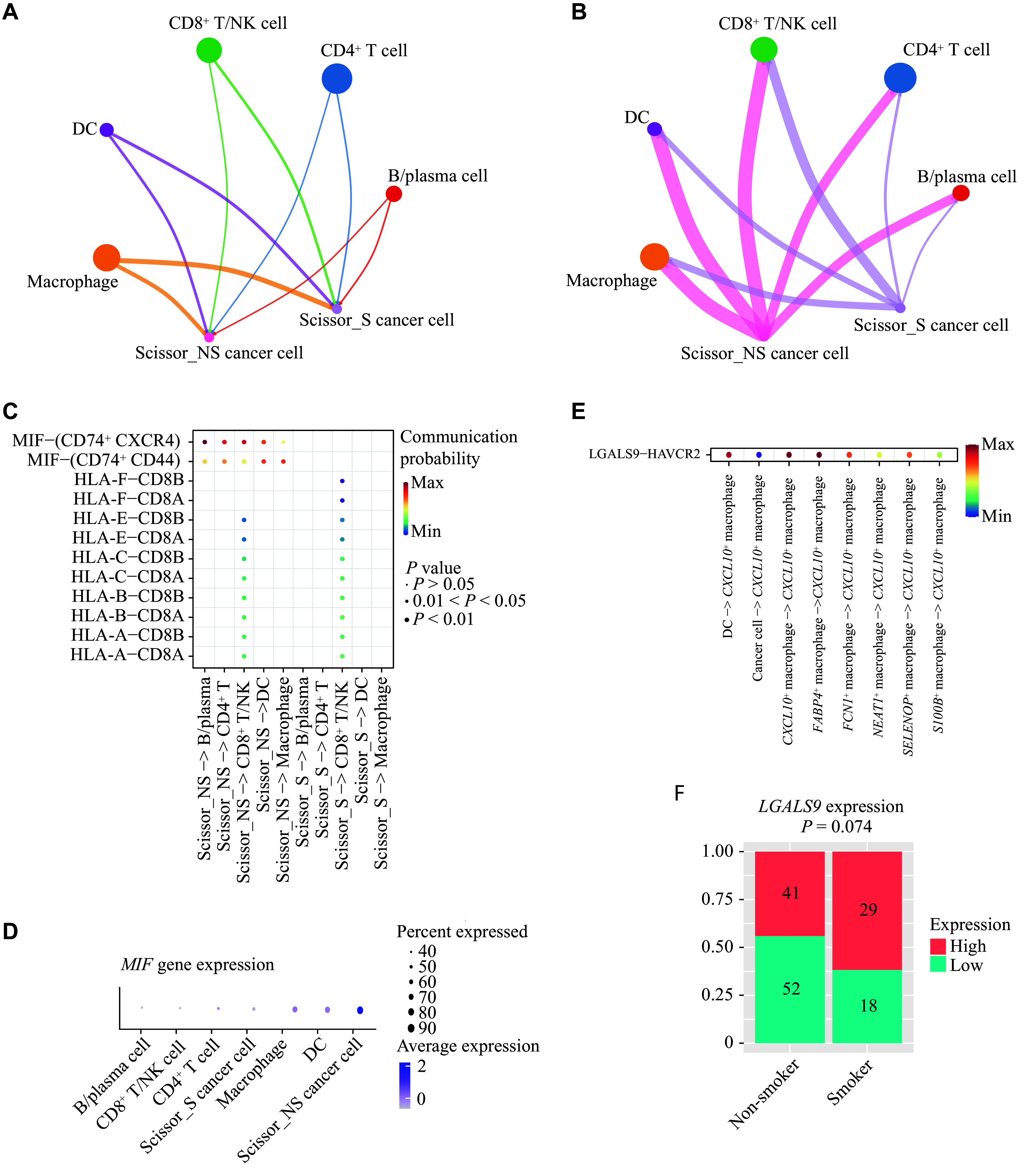
Cell-cell communication analysis. A: Incoming signals to Scissor_S and Scissor_NS cancer cells. A thicker edge line indicates a stronger signal. B: Outgoing signals from Scissor_S and Scissor_NS cancer cells. A thicker edge line indicates a stronger signal. C: Significant interaction associated with MIF and MHC-Ⅰ signaling sent by Scissor_S and Scissor_NS cells. D: *MIF* expression in various cell subtypes. E: Significant LGALS9-HAVCR2 interaction targeting *CXCL10*^+^ macrophages. F: Comparison of bulk *LGALS9* expression in smoking and non-smoking patients. Abbreviations: MHC, major histocompatibility complex; Scissor_NS, non-smoking-associated subpopulations (by the Scissor algorithm); Scissor_S, smoking-associated subpopulations (by the Scissor algorithm).

To clarify the difference in cell-cell communication between LCIS and LCINS, we first compared the interactions of Scissor_S and Scissor_NS cancer cells with other major cell types. The incoming signals to Scissor_S and Scissor_NS cancer cells did not show much difference (***Fig. 6A***), but Scissor_NS cancer cells sent more signals to other cells (***Fig. 6B***). Despite interacting less frequently with other cells, Scissor_S cancer cells still exhibited strong communication signals with CD8^+^ T/NK cells (***Fig. 6B***). This phenomenon may be attributed to the active MHC-Ⅰ–CD8A axis, given the high expression of MHC-Ⅰ molecules on Scissor_S cancer cells (***Figs. 6C*** and ***3H***, and ***Supplementary Fig. 11***). The Scissor_NS cells, on the other hand, were activated in the macrophage migration inhibitory factor (MIF) signaling pathway (***Fig. 6C***), with *MIF* being predominantly expressed in Scissor_NS cancer cells (***Fig. 6D***). Although MIF is a pro-inflammatory molecule, in the context of cancer, it could create a favorable immune microenvironment to facilitate tumor growth^[37]^.

*CXCL10*^+^ macrophages were found to be immunosuppressive and expressed several immune checkpoint molecules, including *PD-L1*, *VSIR*, and *HAVCR2* (***Fig. 4G***). Among these checkpoint molecules, we observed that *CXCL10*^+^ macrophages interacted with several other cell types through the immunoregulatory HAVCR2-LGALS9 axis (***Fig. 6E***). HAVCR2 activation is primarily induced by LGALS9^[38]^, and we noted that *LGALS9* expression was higher in smoking patients (***Fig. 6F***). Therefore, our results suggest that the HAVCR2-LGALS9 axis might contribute to the immunosuppressive properties of *CXCL10*^+^ macrophages in smoking patients.

## Discussion

Our study reveals distinct molecular and immunological landscapes between LUAD in smokers and never-smokers, highlighting distinct oncogenic mechanisms and immune microenvironments that shape different clinical outcomes and therapeutic responses^[3]^. Smoking patients exhibited enhanced tumor aggressiveness, with upregulated pathways linked to cell proliferation and oncogenesis, such as the WNT and MAPK pathways^[39–40]^. These findings align with prior proteomic evidence and are consistent with the established link between smoking and WNT/MAPK pathway activation^[41–42]^. Additionally, smoking-associated LUAD displayed reduced MHC-Ⅱ expression on cancer cells and diminished infiltration of MHC-Ⅱ^+^ immune cells, correlating with reduced CD4^+^ T-cell infiltration, impaired CD4^+^ T-cell activation, and poor prognosis^[5,43–48]^. Smoking-induced AT2 cell disruption—a key population in lung repair—further suggests a mechanism for accelerated tumorigenesis, as AT2 cell loss was associated with poor prognosis^[5]^.

For never-smokers, reduced antitumor cytotoxic activity may contribute to tumorigenesis. Non-smoking patients demonstrated lower expression of MHC-Ⅰ on tumor cells, reduced infiltration of cytotoxic CD8^+^ T and NK cells, and diminished CD8^+^ T-cell cytotoxicity. These characteristics correspond to their lower tumor mutational burden, which correlates with fewer neoantigens and a weaker cytotoxic response, explaining their suboptimal response to immune checkpoint inhibitors^[6,8,49]^. Furthermore, non-smokers exhibited pronounced MIF signaling, which is a proinflammatory cytokine associated with chronic inflammation^[37]^. Previous research has also indicated that prolonged inflammation may reshape the tissue microenvironment and promote lung cancer tumor growth, particularly in non-smoking patients^[50–51]^.

Modulating the tumor microenvironment has emerged as a pivotal focus in contemporary cancer therapy^[52–54]^. As critical orchestrators of the tumor microenvironment, macrophages have become a key area of investigation in both tumor pathogenesis and therapeutic responses^[55–56]^. In the current study, we discovered that *CXCL10*^+^ macrophages were significantly enriched in smoking-associated macrophages. Previous studies have suggested that the infiltration of *CXCL10*^+^ macrophages is associated with a more favorable response to atezolizumab, a PD-L1 inhibitor^[34]^. In our investigation, we also observed the expression of several immune checkpoint molecules in this macrophage subtype, including *PD-L1* and *HAVCR2*. Therefore, the infiltration of *CXCL10*^+^ macrophages in smoking patients may also be associated with a favorable response to immunotherapy^[49]^. Currently, the primary immunotherapeutic approach targets the PD-1–PD-L1 axis. However, patients undergoing anti-PD-1/anti-PD-L1 immunotherapy may develop drug resistance, potentially due to the upregulation of other co-inhibitory molecules, such as HAVCR2^[57]^. We observed significant interaction between *CXCL10*^+^ macrophages and other cells through the HAVCR2-LGALS9 axis, with *LGALS9* found to be upregulated in smoking patients. *In vitro* experiments demonstrated that anti-HAVCR2 antibodies could reverse anti-PD-1 resistance^[58]^. To date, multiple clinical trials evaluating anti-HAVCR2 and anti-LGALS9 therapies have been initiated, and some of them have shown promising results^[58–63]^. Our evidence suggests that targeting the HAVCR2-LGALS9 axis could represent a promising immunotherapy strategy for smoking patients.

Nevertheless, our study has several limitations. Firstly, we employed the Scissor algorithm, which identified smoking-associated and non-smoking-associated cell subpopulations by leveraging bulk phenotype information and the cell-sample correlation matrix. We did not apply Scissor to small cell subgroups, as these subgroups contributed minimally to bulk gene expression, and the results calculated by the Scissor algorithm could be inaccurate. However, these cell subtypes, such as fibroblasts, also play a crucial role in cancer development^[64]^. Secondly, we found increased infiltration of immunosuppressive *CXCL10*^+^ macrophages in LCIS patients through bioinformatic analysis. However, the immunosuppressive properties of *CXCL10*^+^ macrophages require experimental confirmation. Thirdly, future investigations are needed into anti-HAVCR2 and anti-LGALS9 immunotherapy strategies for LUAD patients in both experimental and clinical studies.

In conclusion, we observed that smoking-associated cancer cells displayed increased aggressiveness, which potentially correlates with the diminished survival rates observed in smoking patients. For non-smoking patients, reduced anti-tumor cytotoxicity and the inflammatory microenvironment were identified as potential contributors to their oncogenesis. Additionally, immunosuppressive *CXCL10*^+^ macrophages were found to facilitate tumor progression in smoking patients, and the immunoregulatory LGALS9-HAVCR2 axis emerged as a promising immunotherapeutic target. Our findings illustrate the distinct oncogenic and immune suppression mechanisms in smoking and non-smoking LUAD patients, and present promising immunotherapeutic strategies.

## Additional information

The online version contains supplementary materials available at http://www.jbr-pub.org.cn/article/doi/10.7555/JBR.39.20250160?pageType=en.

## References

[1] (2024). Global cancer statistics 2022: GLOBOCAN estimates of incidence and mortality worldwide for 36 cancers in 185 countries. CA Cancer J Clin.

[2] (2007). Lung cancer in never smokers—a different disease. Nat Rev Cancer.

[3] (2024). Lung cancer in patients who have never smoked—an emerging disease. Nat Rev Clin Oncol.

[4] (2017). Proportion of never-smoker non-small cell lung cancer patients at three diverse institutions. J Natl Cancer Inst.

[5] (2023). Distinct immune microenvironment of lung adenocarcinoma in never-smokers from smokers. Cell Rep Med.

[6] (2021). Genomic profiling of lung adenocarcinoma in never-smokers. J Clin Oncol.

[7] (2018). Lung cancer in never smokers—the East Asian experience. Transl Lung Cancer Res.

[8] (2020). Genomic landscape of lung adenocarcinoma in East Asians. Nat Genet.

[9] (2015). Pathogenic mechanisms of lung adenocarcinoma in smokers and non-smokers determined by gene expression interrogation. Oncol Lett.

[10] (2022). Molecular profiling of human non-small cell lung cancer by single-cell RNA-seq. Genome Med.

[11] (2022). Identifying phenotype-associated subpopulations by integrating bulk and single-cell sequencing data. Nat Biotechnol.

[12] (2021). Inference and analysis of cell-cell communication using CellChat. Nat Commun.

[13] (2020). Single-cell analyses identify dysfunctional CD16^+^ CD8 T cells in smokers. Cell Rep Med.

[14] (2019). Characterizing smoking-induced transcriptional heterogeneity in the human bronchial epithelium at single-cell resolution. Sci Adv.

[15] (2022). High-resolution single-cell atlas reveals diversity and plasticity of tissue-resident neutrophils in non-small cell lung cancer. Cancer Cell.

[16] (2013). STAR: Ultrafast universal RNA-seq aligner. Bioinformatics.

[17] (2020). *ComBat-seq*: Batch effect adjustment for RNA-seq count data. NAR Genom Bioinform.

[18] (2018). An integrated TCGA pan-cancer clinical data resource to drive high-quality survival outcome analytics. Cell.

[19] (2021). Integrated analysis of multimodal single-cell data. Cell.

[b20] 20Korotkevich G, Sukhov V, Budin N, et al. Fast gene set enrichment analysis[PP/OL]. BioRxiv (2021-02-01) [2025-04-01]. https://www.biorxiv.org/content/10.1101/060012v3.

[21] (2005). Gene set enrichment analysis: A knowledge-based approach for interpreting genome-wide expression profiles. Proc Natl Acad Sci U S A.

[22] (2015). The molecular signatures database hallmark gene set collection. Cell Syst.

[23] (2013). GSVA: Gene set variation analysis for microarray and RNA-seq data. BMC Bioinformatics.

[24] (2021). Conserved pan-cancer microenvironment subtypes predict response to immunotherapy. Cancer Cell.

[25] (2019). The single-cell transcriptional landscape of mammalian organogenesis. Nature.

[26] (2021). Deciphering cell lineage specification of human lung adenocarcinoma with single-cell RNA sequencing. Nat Commun.

[27] (2013). Lung adenocarcinoma subtypes based on expression of human airway basal cell genes. Eur Respir J.

[28] (2022). Integrated single-cell RNA sequencing analysis reveals distinct cellular and transcriptional modules associated with survival in lung cancer. Signal Transduct Target Ther.

[29] (2021). Long non-coding RNA ZFAS1 is a major regulator of epithelial-mesenchymal transition through miR-200/ZEB1/E-cadherin, vimentin signaling in colon adenocarcinoma. Cell Death Discov.

[30] (2016). ALDOA functions as an oncogene in the highly metastatic pancreatic cancer. Cancer Lett.

[31] (2024). Single-cell division tracing and transcriptomics reveal cell types and differentiation paths in the regenerating lung. Nat Commun.

[32] (2019). Inflammaging and oxidative stress in human diseases: From molecular mechanisms to novel treatments. Int J Mol Sci.

[33] (2008). IL-10: The master regulator of immunity to infection. J Immunol.

[34] (2023). PD-1^−^ CD45RA^+^ effector-memory CD8 T cells and CXCL10^+^ macrophages are associated with response to atezolizumab plus bevacizumab in advanced hepatocellular carcinoma. Nat Commun.

[35] (2019). The lncRNA Neat1 promotes activation of inflammasomes in macrophages. Nat Commun.

[36] (2004). CD1a and langerin: Acting as more than Langerhans cell markers. J Clin Invest.

[37] (2022). Macrophage migration inhibitory factor (MIF): A multifaceted cytokine regulated by genetic and physiological strategies. Pharmacol Ther.

[38] (1997). Identification and characterization of galectin-9, a novel beta-galactoside-binding mammalian lectin. J Biol Chem.

[39] (2021). Mutations and mechanisms of WNT pathway tumour suppressors in cancer. Nat Rev Cancer.

[40] (2019). MAPK pathway: A potential target for the treatment of non-small-cell lung carcinoma. Future Med Chem.

[41] (2023). Cigarette smoking induces lung cancer tumorigenesis *via* upregulation of the WNT/β-catenin signaling pathway. Life Sci.

[42] (2022). Cigarette smoke extract-mediated FABP4 upregulation suppresses viability and induces apoptosis, inflammation and oxidative stress of bronchial epithelial cells by activating p38 MAPK/MK2 signaling pathway. J Inflamm (Lond).

[43] (2019). Biological consequences of MHC-Ⅱ expression by tumor cells in cancer. Clin Cancer Res.

[44] (2004). Function and regulation of MHC class Ⅱ molecules in T-lymphocytes: Of mice and men. Hum Immunol.

[45] (2020). Cancer cell-intrinsic expression of MHC class Ⅱ regulates the immune microenvironment and response to anti-PD-1 therapy in lung adenocarcinoma. J Immunol.

[46] (2017). Expression of the MHC class Ⅱ in triple-negative breast cancer is associated with tumor-infiltrating lymphocytes and interferon signaling. PLoS One.

[47] (2024). CD4^+^ T cell immunity against cutaneous melanoma encompasses multifaceted MHC Ⅱ-dependent responses. Sci Immunol.

[48] (2008). Increased HLA-DMB expression in the tumor epithelium is associated with increased CTL infiltration and improved prognosis in advanced-stage serous ovarian cancer. Clin Cancer Res.

[49] (2019). Predictors for clinical benefit of immune checkpoint inhibitors in advanced non-small-cell lung cancer: A meta-analysis. Immunotherapy.

[50] (2018). Whole-genome sequencing reveals genomic signatures associated with the inflammatory microenvironments in Chinese NSCLC patients. Nat Commun.

[51] (2023). Lung cancer immunotherapy: Progress, pitfalls, and promises. Mol Cancer.

[52] (2021). Therapeutic targeting of the tumor microenvironment. Cancer Discov.

[53] (2024). Non-invasive physical stimulation to modulate the tumor microenvironment: Unveiling a new frontier in cancer therapy. BIO Integr.

[54] (2024). Voluntary exercise sensitizes cancer immunotherapy *via* the collagen inhibition-orchestrated inflammatory tumor immune microenvironment. Cell Rep.

[55] (2023). Targeted imaging of tumor associated macrophages in breast cancer. BIO Integr.

[56] (2023). PTBP2-mediated alternative splicing of IRF9 controls tumor-associated monocyte/macrophage chemotaxis and repolarization in neuroblastoma progression. Research (Wash D C).

[57] (2016). Adaptive resistance to therapeutic PD-1 blockade is associated with upregulation of alternative immune checkpoints. Nat Commun.

[58] (2019). Tim-3/galectin-9 pathway and mMDSC control primary and secondary resistances to PD-1 blockade in lung cancer patients. Oncoimmunology.

[59] (2023). A new emerging target in cancer immunotherapy: Galectin-9 (LGALS9). Genes Dis.

[b60] 60A phase 1, open-label, multicenter trial investigating the safety, tolerability, and preliminary antineoplastic activity of Sym023 (anti-TIM-3) in patients with advanced solid tumor malignancies or lymphomas[EB/OL]. [2018-04-05]. https://clinicaltrials.gov/study/NCT03489343.

[b61] 61TIM3 inhibition with MBG453 for patients with lower risk MDS: An adaptive two-stage phase Ⅱ clinical trial[EB/OL]. [2025-04-01]. https://www.dana-farber.org/clinical-trials/20-637.

[62] (2021). Phase Ⅰ/Ⅰb clinical trial of sabatolimab, an anti-TIM-3 antibody, alone and in combination with spartalizumab, an anti-PD-1 antibody, in advanced solid tumors. Clin Cancer Res.

[63] (2018). A phase 1 study of TSR-022, an anti-TIM-3 monoclonal antibody, in combination with TSR-042 (anti-PD-1) in patients with colorectal cancer and post-PD-1 NSCLC and melanoma. J Immunother Cancer.

[64] (2023). Single-cell analysis reveals prognostic fibroblast subpopulations linked to molecular and immunological subtypes of lung cancer. Nat Commun.

