# Supplementary material for: Integrating bulk and single-cell sequencing reveals cellular heterogeneity between lung adenocarcinoma in smokers and never-smokers
Source: J Biomed Res. 2026 May 21;40(3):247–65. doi: 10.7555/JBR.39.20250160 (PMC13231364; doi:10.7555/JBR.39.20250160)
Supplement: Supplementary file 1 — The online version contains supplementary materials available at http://www.jbr-pub.org.cn/article/doi/10.7555/JBR.39.20250160?pageType=en. [file jbr-40-3-247-S1.pdf]

Available online at www.jbr-pub.org.cn

Open Access at PubMed Central

Journal of Biomedical Research, 2026 40(3): 1–8

Supplementary Data

JBR

Integrating bulk and single-cell sequencing reveals cellular heterogeneity between lung adenocarcinoma in smokers and never-smokers

Zihuan Zhao[1,△], Pan Yang[2,△], Yuhan Liu[1,△], Kai Wang[1,3], Xianfeng Xu[1], Yuzhuo Wang[1], Meng Zhu[2], Na Qin[1], Cheng Wang[1], Weimin Li[2], Hongxia Ma[1,✉], Zhoufeng Wang[2,✉], Hongbing Shen[1,4,✉]

[1]*Department of Epidemiology, Center for Global Health, School of Public Health, Nanjing Medical University, Nanjing, Jiangsu 211166, China;*
[2]*Institute of Respiratory Health, Frontiers Science Center for Disease-related Molecular Network, West China Hospital, Sichuan University, Chengdu, Sichuan 610041, China;*
[3]*Department of Epidemiology, School of Public Health, Southeast University, Nanjing, Jiangsu 210096, China;*
[4]*Research Units of Cohort Study on Cardiovascular Diseases and Cancers, Chinese Academy of Medical Sciences, Beijing 100037, China.*

Supplementary Table 1 **Baseline characteristics of 140 patients with bulk RNA-seq data**

Characteristics	Non-smokers (n = 93)	Smokers (n = 47)	P value
Age group (years, n [%])			0.532
≤ 65	63 (67.74)	35 (74.47)	
> 65	30 (32.26)	12 (25.53)	
Sex (n [%])			< 0.001
Male	23 (24.73)	47 (100.00)	
Female	70 (75.27)	0 (0)	
Stage (n [%])			0.985
Early (Ⅰ – Ⅰ)	61 (65.59)	30 (63.83)	
Advanced (Ⅲ–Ⅳ)	32 (34.41)	17 (36.17)	
Source (n [%])			0.544
NJLCC	54 (58.06)	24 (51.06)	
WCH	39 (41.94)	23 (48.94)	

P value was derived from the Chi-squared test. Abbreviations: NJLCC, Nanjing Lung Cancer Cohort; WCH, West China Hospital.

△These authors contributed equally to this work.

✉Corresponding authors: Hongxia Ma, E-mail: hongxiama@njmu.edu.cn (ORCID: 0000-0002-9821-6955); Zhoufeng Wang, E-mail: wangzhoufeng@scu.edu.cn (ORCID: 0000-0002-7881-1837); Hongbin Shen, E-mail: hbshen@njmu.edu.cn (ORCID: 0000-0002-2581-5906).

Received: 15 April 2025; Revised: 18 August 2025; Accepted: 25 August 2025; Available online: 25 August 2025; Published date: 21 May 2026

CLC number: R734, Document code: A

The corresponding author Hongbing Shen was a former editor-in-chief of this journal but had no involvement in the editorial handling or peer review process of this manuscript. The authors reported no conflict of interests.

This is an open access article under the Creative Commons Attribution (CC BY 4.0) license, which permits others to distribute, remix, adapt and build upon this work, for commercial use, provided the original work is properly cited.

© 2026 by Journal of Biomedical Research.

https://doi.org/10.7555/JBR.39.20250160

Supplementary Table 2 **Baseline characteristics of 20 patients with scRNA-seq data**

Characteristics	Non-smokers (n = 7)	Smokers (n = 13)	P value
Sex (n [%])			0.031
Female	4 (57.14)	1 (7.69)	
Male	3 (42.86)	12 (92.31)	
Stage (n [%])			0.354
Early (Ⅰ–Ⅱ)	6 (85.71)	8 (61.54)	
Advanced (Ⅲ–Ⅳ)	1 (14.29)	5 (38.46)	
Study (n [%])			0.29
He_Fan_2021	3 (42.86)	2 (15.38)	
Kim_Lee_2020	4 (57.14)	11 (84.62)	

P value was derived from the Fisher's exact test.

Supplementary Table 3. Differential gene expression between Scissor_S and Scissor_NS cells (available online)

Supplementary Table 4 Differential gene expression and pathway analysis between Scissor_S and Scissor_NS cancer cells (available online)

Supplementary Table 5 Differential gene expression and pathway analysis of *CXCL10*⁺ macrophages and *NEAT1*⁺ macrophages (available online)

Supplementary Table 6 Differential gene expression and pathway analysis of *CXCL10*⁺ DCs, plasmacytoid DCs, and *CD1A*⁺ DCs (available online)

Supplementary Table 7 Differential gene expression and pathway analysis of NK/CTL and *GZMK*⁺CD8⁺ T cells (available online)

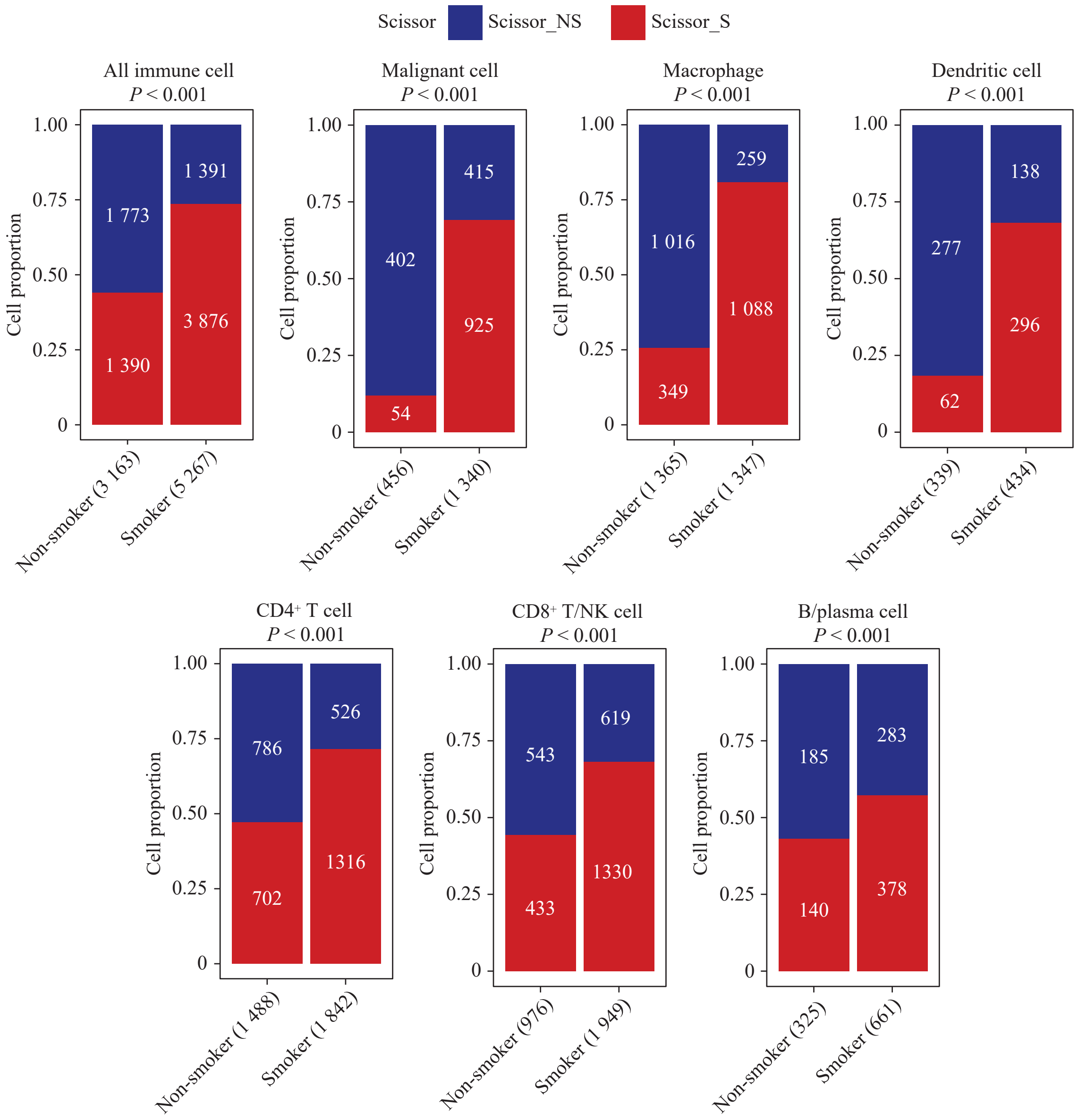

Supplementary Fig. 1 **Distribution of Scissor-selected cells in smokers and non-smokers.** The proportion of Scissor S/NS cells in smoking and non-smoking patients was compared using the Chi-squared test. Numbers in parentheses represent the counts of Scissor-selected cells in smokers and non-smokers of the scRNA-seq dataset. Abbreviations: Scissor_NS, non-smoking-associated subpopulations (by the Scissor algorithm); Scissor_S, smoking-associated subpopulations (by the Scissor algorithm).

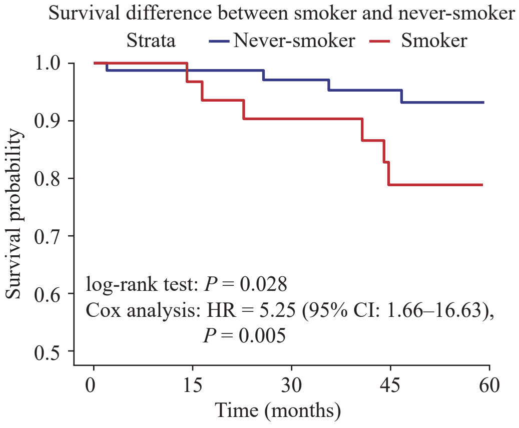

Supplementary Fig. 2 **Survival curve and Cox regression analysis between smoking and non-smoking LUAD patients.** Tumor stage was adjusted in the multivariate Cox regression model. Abbreviations: CI: confidence interval; HR, hazard ratio; LUAD, lung adenocarcinoma.

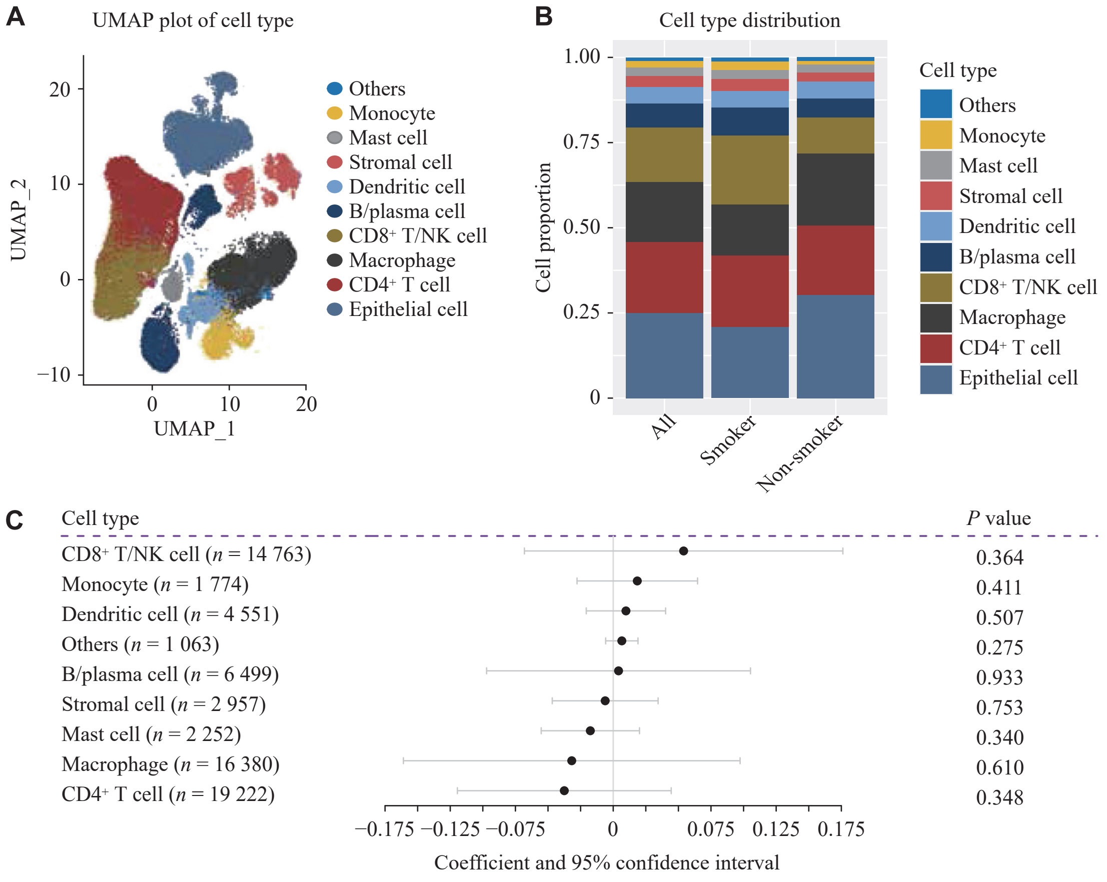

Supplementary Fig. 3 **Cellular landscape of East Asian LUAD patients.** A: UMAP plot of all included cells, colored by cell subtypes. B: Barplot of cell subtype proportion of all patients, and in smoking and non-smoking subgroups. C: Linear regression model comparing cell subtype proportions, adjusted by different datasets. Abbreviations: LUAD, lung adenocarcinoma; UMAP, uniform manifold approximation and projection.

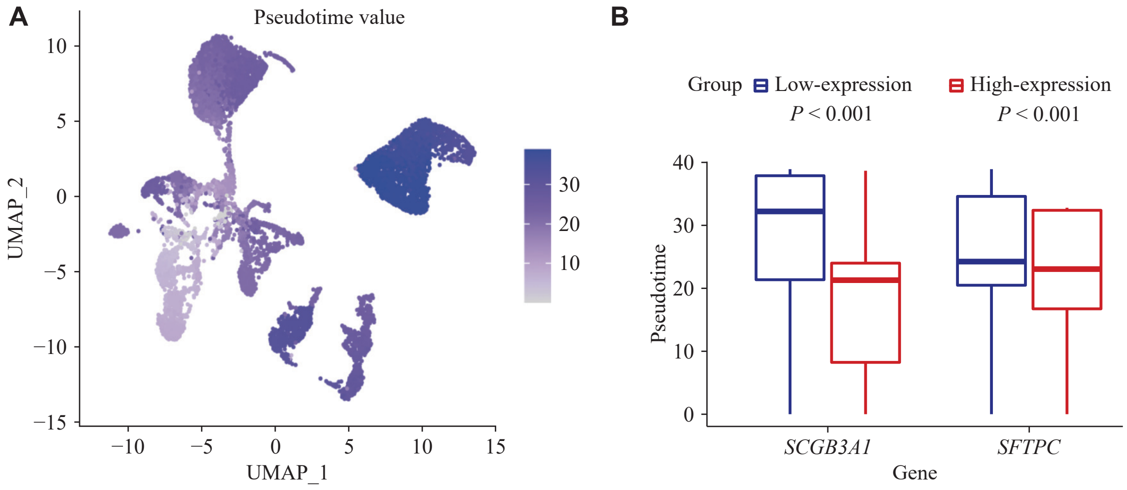

Supplementary Fig. 4 **Trajectory analysis of cancer cells.** A: UMAP plot of cancer cells, colored by the pseudotime value. B: Associations between differentiation markers expression and pseudotime values. Cancer cells were divided into high and low groups, based on median expression values of *SCGB3A1* and *SFTPC* genes, and *P* value was calculated by the two-tailed Wilcoxon rank sum test. Abbreviation: UMAP, uniform manifold approximation and projection.

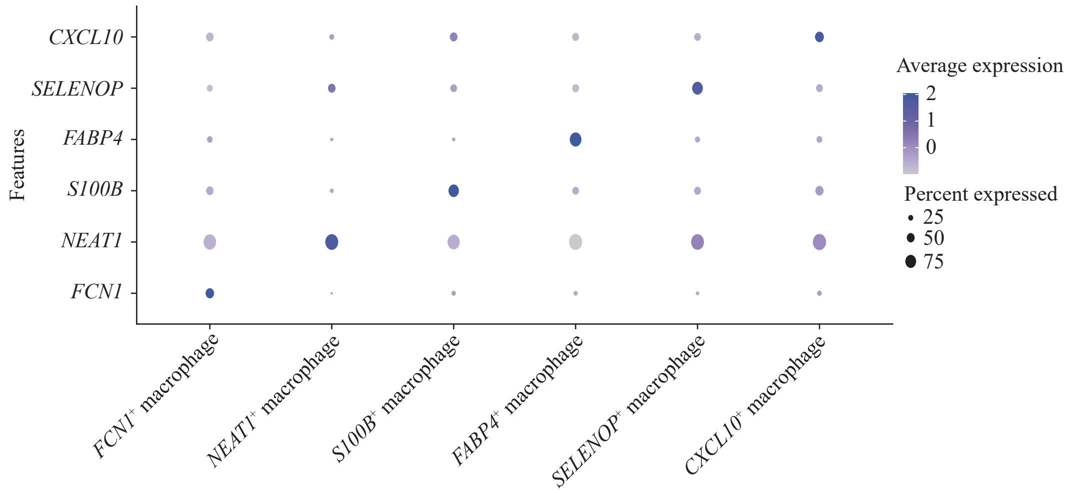

Supplementary Fig. 5 **Dot plot showing the expression of marker genes in each macrophage subtype.**

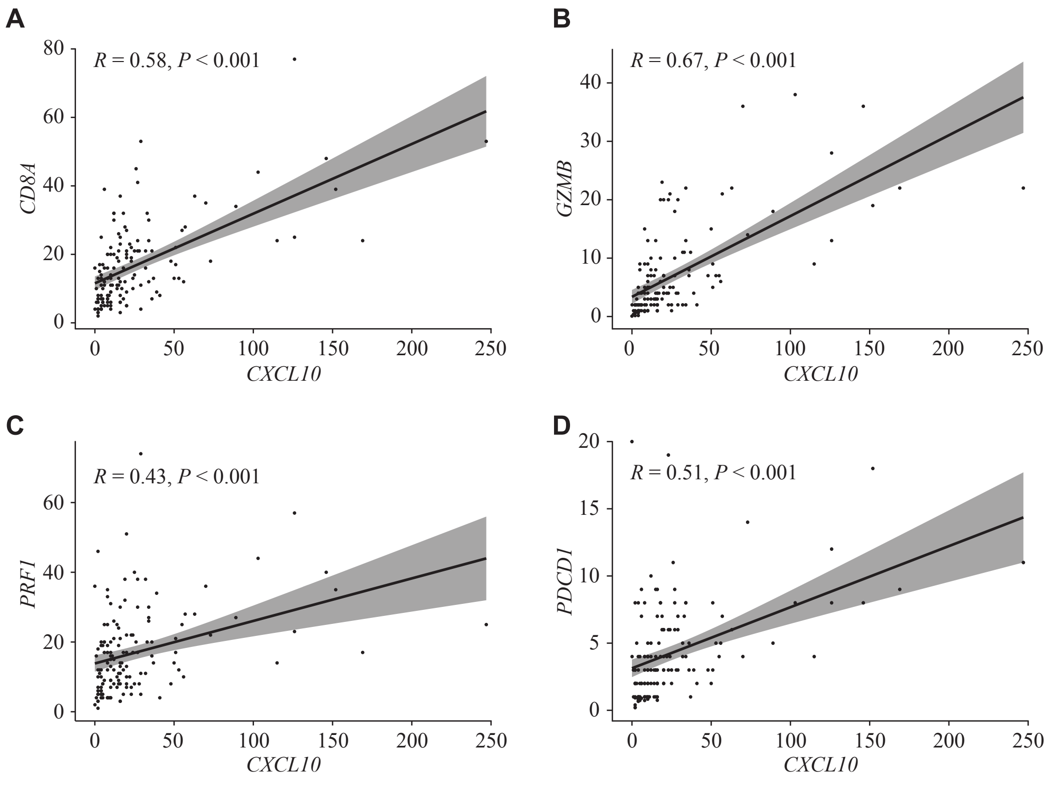

Supplementary Fig. 6 **Correlation of *CXCL10* with *CD8A* (A), cytotoxic molecule *GZMB* (B), cytotoxic molecule *PRF1* (C), and *PDCD1* (D).**

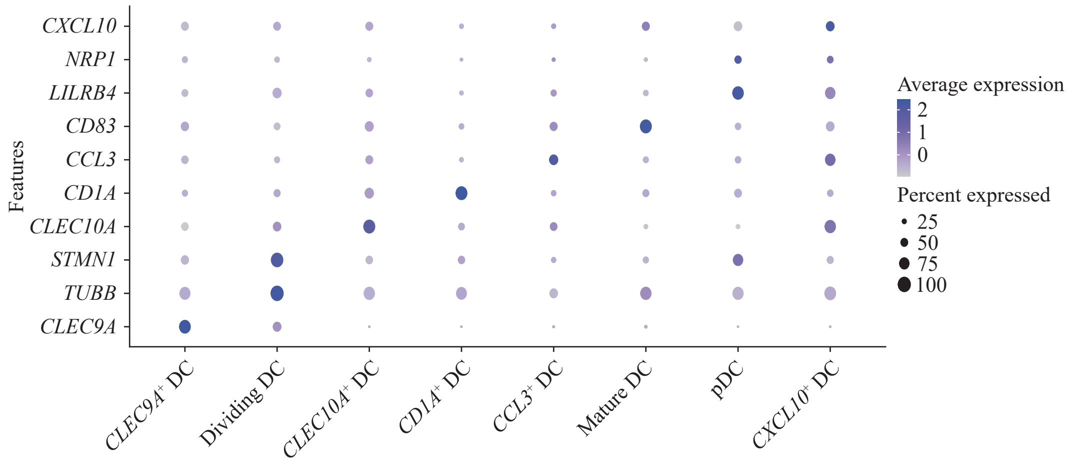

Supplementary Fig. 7 **Dot plot showing the expression of marker genes in each dendritic cell (DC) subtype.**

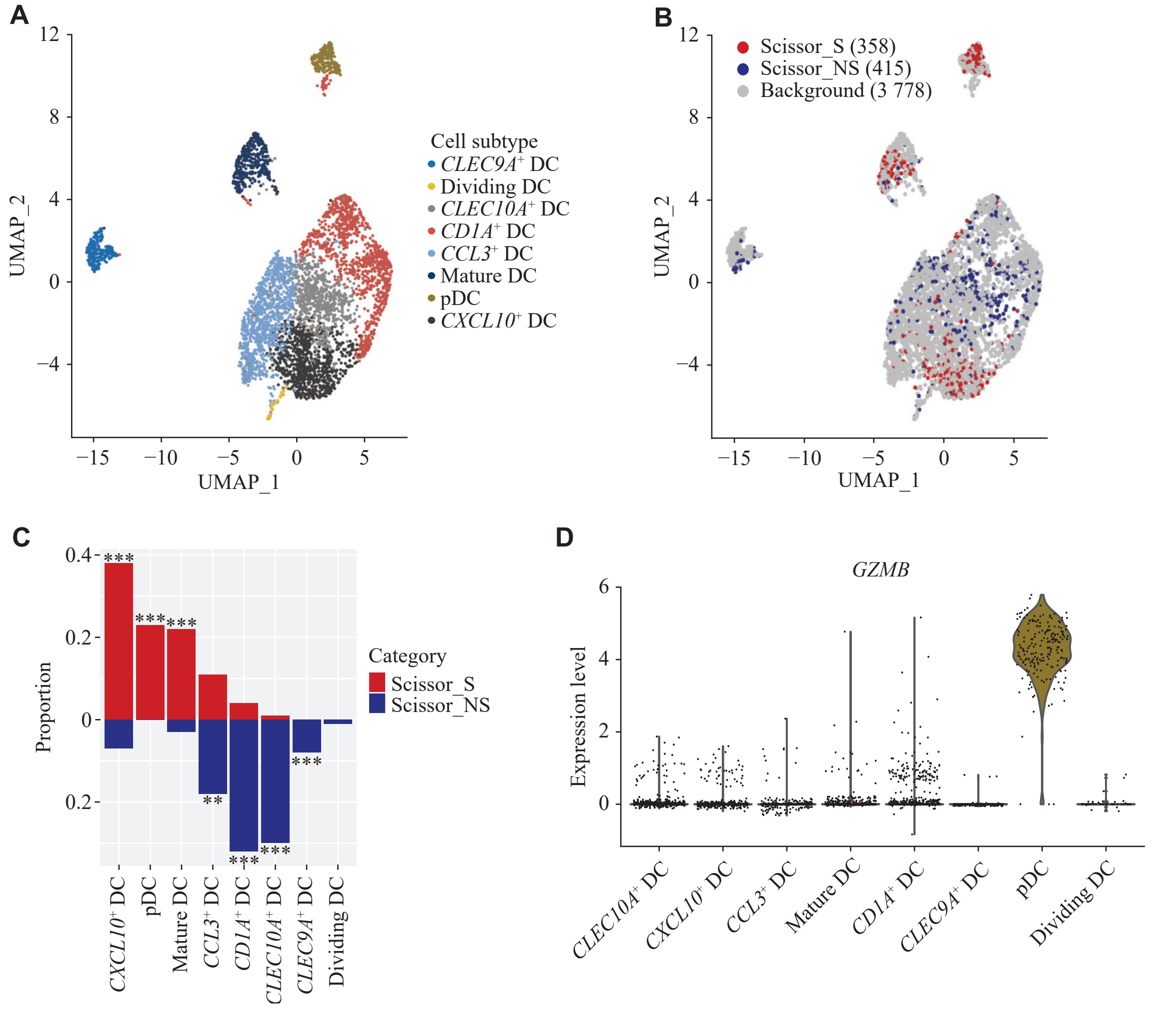

Supplementary Fig. 8 **Characterization of smoking-associated and non-smoking-associated dendritic cell (DC) subpopulations.** A: UMAP plot of dendritic cells, colored by cell subtypes. B: UMAP plot of Scissor-selected smoking-associated and non-smoking-associated DC subpopulations. C: Comparison of cell subtype proportions in Scissor_S and Scissor_NS DCs.**FDR < 0.01, and ***FDR < 0.001. D: *GZMB* expression in different DC cell subtypes. Abbreviations: FDR, false discovery rate; pDC, plasmacytoid dendritic cell; Scissor_NS, non-smoking-associated subpopulations (by the Scissor algorithm); Scissor_S, smoking-associated subpopulations (by the Scissor algorithm); UMAP, uniform manifold approximation and projection.

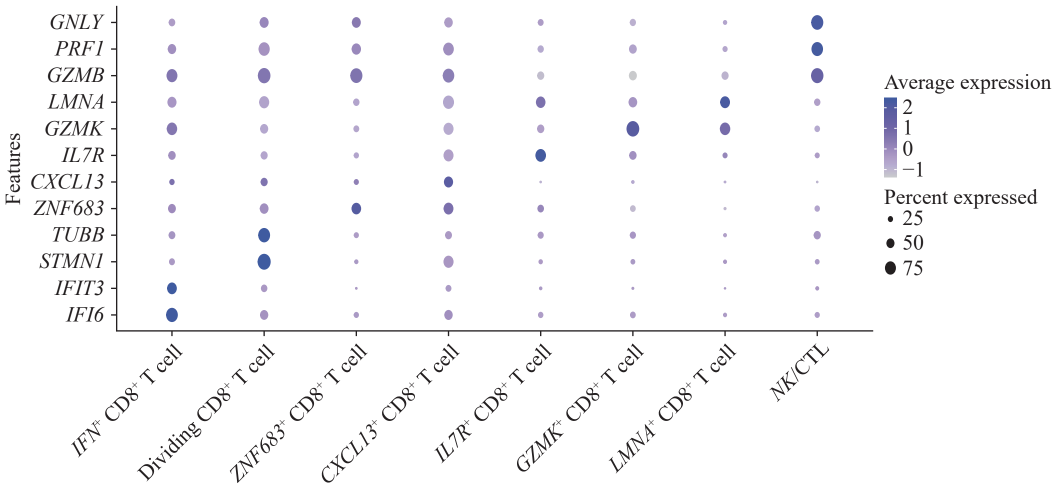

Supplementary Fig. 9 **Dot plot showing the expression of marker genes in each CD8+ T/NK cell subtype.**

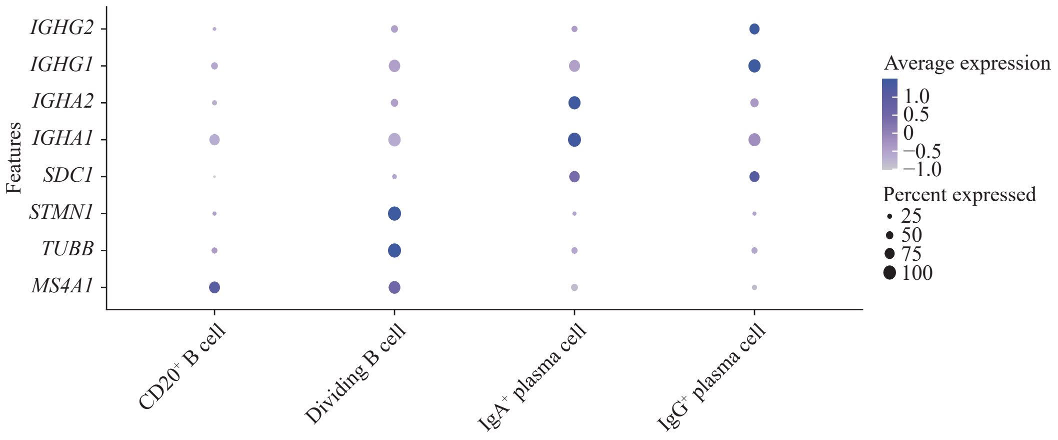

Supplementary Fig. 10 **Dot plot showing the expression of marker genes in each B/plasma cell subtype.**

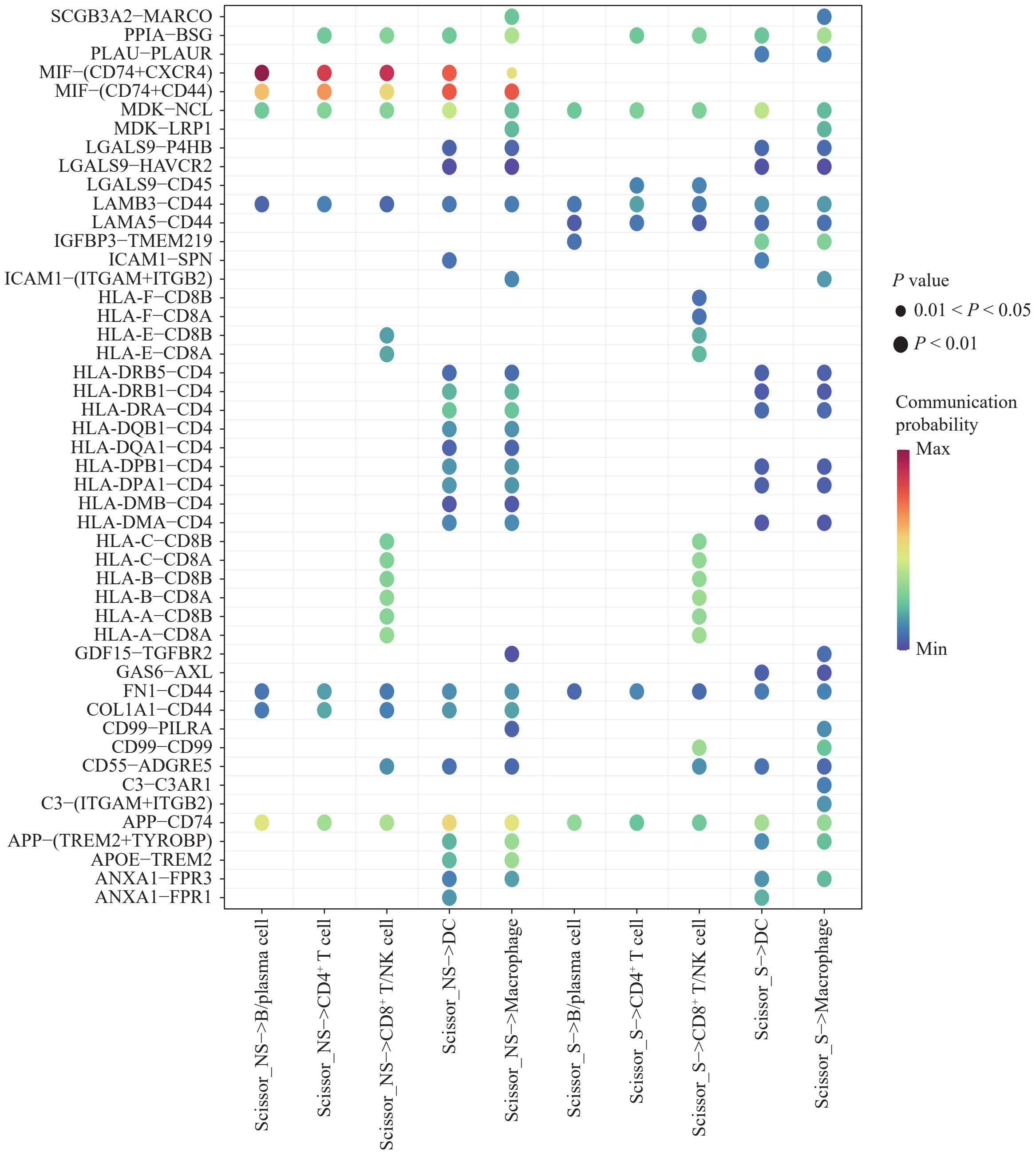

Supplementary Fig. 11 **Significant interactions sent by Scissor_S and Scissor_NS cancer cells.** Abbreviations: Scissor_NS, non-smoking-associated subpopulations (by the Scissor algorithm); Scissor_S, smoking-associated subpopulations (by the Scissor algorithm).
